# Synthetic cannabinoid receptor agonists are monoamine oxidase‐A selective inhibitors

**DOI:** 10.1111/febs.16741

**Published:** 2023-02-21

**Authors:** Sarah A. Hindson, Rachael C. Andrews, Michael J. Danson, Marc W. van der Kamp, Amy E. Manley, Oliver B. Sutcliffe, Tom S. F. Haines, Tom P. Freeman, Jennifer Scott, Stephen M. Husbands, Ian S. Blagbrough, J. L. Ross Anderson, David R. Carbery, Christopher R. Pudney

**Affiliations:** ^1^ Department of Biology and Biochemistry University of Bath BA2 7AY Bath UK; ^2^ Department of Chemistry University of Bath BA2 7AY Bath UK; ^3^ Centre for Sustainable and Circular Technologies University of Bath BA2 7AY Bath UK; ^4^ School of Biochemistry University of Bristol BS8 1TD Bristol UK; ^5^ Faculty of Health Sciences University of Bristol BS8 1TH Bristol UK; ^6^ MANchester DRug Analysis & Knowledge Exchange (MANDRAKE), Department of Natural Sciences Manchester Metropolitan University M15 5GD Manchester UK; ^7^ Department of Computer Science University of Bath BA2 7AY Bath UK; ^8^ Department of Psychology University of Bath BA2 7AY Bath UK; ^9^ Department of Pharmacy and Pharmacology University of Bath BA2 7AY Bath UK; ^10^ Centre for Therapeutic Innovation University of Bath BA2 7AY Bath UK

**Keywords:** enzyme inhibition, flexible docking analysis, MAO‐A, MAO‐B, monoamine oxidase, SCRAs, synthetic cannabinoid receptor agonists

## Abstract

Synthetic cannabinoid receptor agonists (SCRAs) are one of the fastest growing classes of recreational drugs. Despite their growth in use, their vast chemical diversity and rapidly changing landscape of structures make understanding their effects challenging. In particular, the side effects for SCRA use are extremely diverse, but notably include severe outcomes such as cardiac arrest. These side effects appear at odds with the main putative mode of action, as full agonists of cannabinoid receptors. We have hypothesized that SCRAs may act as MAO inhibitors, owing to their structural similarity to known monoamine oxidase inhibitors (MAOI's) as well as matching clinical outcomes (hypertensive crisis) of ‘monoaminergic toxicity’ for users of MAOIs and some SCRA use. We have studied the potential for SCRA‐mediated inhibition of MAO‐A and MAO‐B via a range of SCRAs used commonly in the UK, as well as structural analogues to prove the atomistic determinants of inhibition. By combining *in silico* and experimental kinetic studies we demonstrate that SCRAs are MAO‐A‐specific inhibitors and their affinity can vary significantly between SCRAs, most notably affected by the nature of the SCRA ‘head’ group. Our data allow us to posit a putative mechanism of inhibition. Crucially our data demonstrate that SCRA activity is not limited to just cannabinoid receptor agonism and that alternative interactions might account for some of the diversity of the observed side effects and that these effects can be SCRA‐specific.

## Graphical abstract

We have studied the potential for SCRA‐mediated inhibition of MAO‐A and MAO‐B using a range of SCRAs used commonly in the UK, as well as structural analogues to prove the atomistic determinants of inhibition. By combining *in silico* and experimental kinetic studies, we demonstrate that SCRAs are MAO‐A‐specific inhibitors, and the affinity can vary significantly between SCRAs, with a mechanism posited.

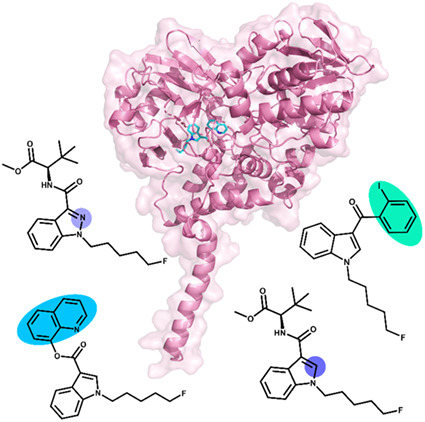

**Abbreviations**
5F‐ADB: N‐[[1‐(5‐fluoropentyl)‐1H‐indazol‐3‐yl]carbonyl]‐3‐methyl‐d‐valine methyl ester
5F‐MDMB‐PICA: methyl‐2‐[[1‐(5‐fluoropentyl)indole‐3‐carbonyl]amino]‐3,3‐dimethyl‐butanoate
5F‐PB‐22: quinolin‐8‐yl 1‐pentyfluoro‐1H‐indole‐3‐8‐carboxylate
AM‐2201: 1‐(5‐fluoropentyl)‐3‐(1‐naphthoyl)indole
AM‐694: 1‐(5‐fluoropentyl)‐3‐(2‐iodobenzoyl)indole
CB1: cannabinoid receptor type 1
CB2: cannabinoid receptor type 2
IC50: half‐maximal inhibitory concentration
MAO: monoamine oxidase
SCRA: synthetic cannabinoid receptor agonist
THC: tetrahydrocannabinol

## Introduction

Synthetic cannabinoid receptor agonists (SCRAs), commonly referred to as ‘spice’ or ‘K2’, are the most rapidly growing class of recreational drugs [1, 2, 3]. These compounds were originally developed for research purposes as SCRAs bind to the cannabinoid receptors CB1 and CB2, mimicking the effect of tetrahydrocannabinol (THC), the main psychoactive component of Cannabis [4, 5, 6, 7, 8]. The cannabinoid receptor interaction with THC has been well studied, with CB1, present in the brain and central nervous system, responsible for the psychoactive effects, and CB2 involved with the immune system [5, 9, 10, 11]. THC only shows partial agonism for the CB receptors, whereas SCRAs are typically high‐affinity full agonists making them highly potent and often unpredictable in comparison [5, 8, 12, 13, 14]. The using community, at least in the UK, is primarily homeless people and people in prisons [2, 13, 15, 16]. The nature of the using population and their circumstance thus presents significant challenges to harm reduction and intervention strategies.

**Fig. 1:**
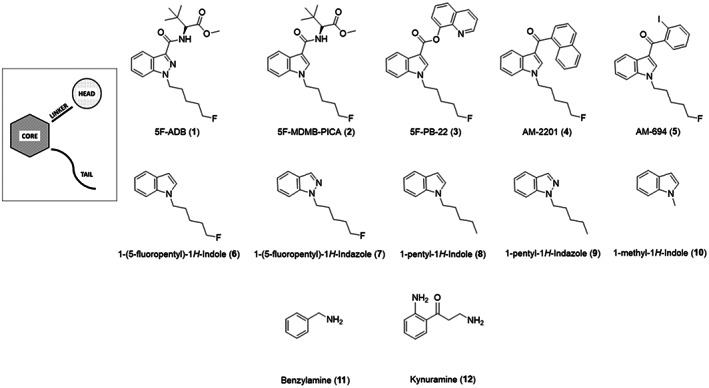
Structures of compounds investigated in this study. (A) Schematic of general SCRA architecture and alterations made to create SCRA derivatives studies. (B) SCRA structures; These compounds include synthetic cannabinoid receptor agonists (**1**–**5**), five compounds that emulate the core and tail section of SCRAs (**6**–**10**), benzylamine **11**, and kynuramine **12**.

In order to circumvent legislation, manufacturers are structurally diversifying the SCRA compounds they synthesise by introducing ‘scaffold hopping’ into their drug design [13, 17, 18, 19]. Essentially, they are able to produce families of novel compounds that share similar structures but are able to mitigate some of the legal restrictions that are in place around the world. The common architecture of spice compounds consists of a ‘head’, ‘linker’, ‘core’ and ‘tail’ group that can be substituted to introduce structural variety (Fig. 1) [2, 7, 13, 17]. As a result, the interactions of these SCRA libraries with biological targets can vary immensely [8, 13, 18, 19, 20]. However, it is clear that fatal side effects from SCRA consumption have been reported across a broad range of ‘spice’ compounds [13, 21].

SCRA consumption frequently leads to severe and adverse health effects compared to those seen from cannabis usage [1, 3, 5, 18, 21]. These include tachycardia, hypertension, myocardial infarction, stroke, acute kidney injury and cardiac arrest to name a few. The origin of such side effects is not well understood, as there is a distinct lack of evidence around the pharmacological and toxicological effects of these compounds. However, such side effects are not obviously associated with CB1/2 agonism [5, 13].

We hypothesised that given some of the side effects of SCRA consumption do not track with CB1/2 agonism, there may be alternative biological interactions. SCRAs have structures which are reminiscent of some monoamine‐oxidase inhibitors. Indeed, MAO assays from pig brain isolates have shown that WIN,55,212‐2 inhibits MAO‐A with an IC_50_ of 18 μm [22]. Monoamine oxidase (MAO) enzymes catalyse the oxidative deamination of ‘biogenic amines’ including key neurotransmitters in the brain [23, 24, 25, 26]. The two MAO isoforms, MAO‐A and MAO‐B, are structurally very similar but have slightly differing substrate specificities, with MAO‐A favouring noradrenaline, adrenaline, serotonin and dopamine, and MAO‐B, β‐phenylethylamine, benzylamine and dopamine also [24, 25, 26, 27, 28]. As such, MAO enzymes pose an attractive drug target in the treatment of neurodegenerative disorders, with much research focussing on the design of MAO inhibitors (MAOI) [29, 30, 31, 32, 33].

A number of MAO‐I drugs exist, but their use can be associated with hypertensive and cardiac effects that result from adrenergic toxicity [27, 33, 34, 35, 36, 37]. The so‐called ‘tyramine pressor response’ occurs under high concentrations of dietary tyramine, which can arise from specific foods including cheese, dried meats and beer [27]. The pressor response is primarily associated with MAO‐A [27, 29]. Consequently, patients taking MAO‐I's are instructed to monitor their blood pressure and follow restricted diets to avoid such ‘monoaminergic toxicity’ [27, 34, 36]. Given that the pressor response can give rise to symptoms similar to some of the ‘unexplained’ symptoms of SCRA use including hypertension and stroke, and SCRAs have structural similarity to known MAO‐Is, we test the hypothesis that SCRAs might act as MAO‐Is. Herein, we study the effect of a range of commonly abused SCRAs on inhibition of MAO‐A and MAO‐B in order to explain the severe hypertensive side effects associated with this class of drug. We use a synthetic organic chemical approach to dissect the molecular determinants of inhibition and are able to report upon the inhibitory effect of a number of SCRAs on MAO activity both *in silico* and *in vitro*.

## Results and Discussion

### *In silico* docking studies identify different binding strengths and modes between SCRAS and MAO‐A/B

To investigate the atomistic determinants of the potential inhibitory effects of synthetic cannabinoids on monoamine oxidases, we have turned to *in silico* docking studies. We have opted for an *in silico* approach since crystallisation of MAOs is notoriously challenging and *in silico* docking studies have been fruitfully used in the study of MAO‐Is previously [38]. The ligands used in the docking analysis (Fig. 1) include five SCRAs, 5F‐ADB **1**, 5F‐MDMB‐PICA **2**, 5F‐PB‐22 **3**, AM‐2201 **4**, and AM‐694 **5**. These compounds have been chosen due to their regular presence in SCRA seizures [18, 21, 39, 40]. Five other compounds **6**–**10** were also chosen containing either an indazole or indole core group. These were used to investigate the effect of the head, core and tail sections on the putative monoamine oxidase inhibition.

X‐ray crystal structures of MAO‐A (PDB: 2Z5X) and MAO‐B (PDB: 2V5Z) were obtained from the Protein Data Bank and prepared in autodock 4.2. (Centre for Computational Structural Biology, The Scripps Research Institute, La Jolla, CA, USA) The ligands bound into the crystal structure were removed alongside all water molecules, while polar hydrogens were added. Only chain A of the MAO‐B structure was used in the docking calculations for computational simplicity. The ligand chemical structures were drawn on chem3d 16.0 (PerkinElmer Informatics, Waltham, MA, USA) software and optimised with DFT. Flexible docking was then undertaken using autodock vina [41], selecting specific residues in the protein active site and labelling them as flexible. All other residues remained rigid.

Initially, a validation study was carried out using an identical docking method, with the co‐crystallised inhibitors from the original pdb files; harmine into MAO‐A and safinamide into MAO‐B. The lowest energy output conformations were compared to the original ligand conformation (Fig. S1). The simulated and crystal harmine ligands have an RMSD value of 1.237 Å and the safinamide ligands have an RMSD value of 0.965 Å, calculated using dockrmsd software [42]. This is below the accepted limit of 2.0 Å for RMSD scoring [38], validating the approach for use with the SCRAs and analogues.

**Fig. 2:**
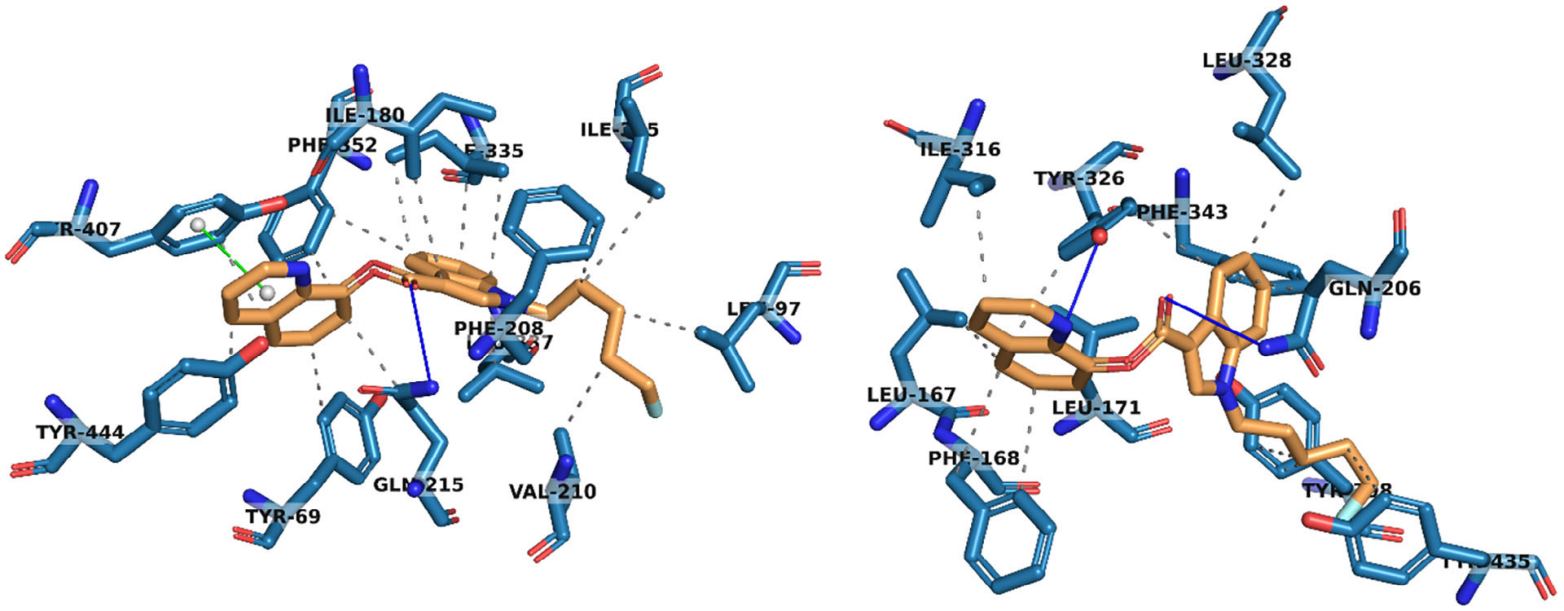
3D protein interactions of 5F‐PB‐22 (**3**) with residues in the active site of MAO‐A (left) and MAO‐B (right) from docking studies. Hydrophobic interactions have been represented with dashed grey lines, hydrogen bonds with solid blue lines and pi‐stacking interactions with solid green lines. Structure figures were generated using pymol (The PyMOL Molecular Graphics System, Version 2.4.1, Schrödinger, LLC, New York, NY, USA).

Compounds **1**–**12** (Fig. 1) were docked into both MAO‐A and MAO‐B using autodock vina, and the lowest docking scores from the 9 output modes of each ligand/protein combination are given in Table 1. Although *in‐silico* docking scores are not able to predict true binding affinities, these values allow us to compare probable protein‐ligand interactions with a range of ligands. Benzylamine (BZA) **11** and kynuramine (KYN) **12** are biogenic amines that are broken down by MAO‐A and MAO‐B, utilising the FAD co‐factor [24, 43]. These two compounds were also included in the docking study as comparative natural binding substrates for the MAO proteins. The non‐covalent binding interactions within the docked protein‐ligand complexes have been analysed using Protein‐Ligand Interaction Profiler (plip) software [44], with the results given in Table 1. Figure 2 also shows an example of these binding interactions, displaying all predicted non‐covalent interactions between 5F‐PB‐22 **3** and residues within the active site of MAO‐A and MAO‐B. All other ligand‐protein interactions can be seen in Figs S2 and S3.

**Table 1:** MAO *in silico* binding results.

Compound	MAO‐A				MAO‐B			
	Docking score (binding free energy/kcal·Mol^−1^)	Hydrophobic interactions	Hydrogen bonds	π‐Stacking interactions	Docking score (binding free energy/kcal·Mol^−1^)	Hydrophobic interactions	Hydrogen bonds	π‐Stacking interactions
1	−9.0	11			−8.8	13		
2	−9.0	11			−8.8	14		
3	−10.6	15	1	1	−10.7	13	2	
4	−10.1	11			−11.0	9	1	
5	−9.4	14	1		−9.2	10	1	
6	−7.8	9			−7.6	5		
7	−7.7	8	1		−7.5	5		
8	−7.8	7			−7.6	8		
9	−7.8	10			−7.5	6		
10	−6.6	6			−6.4	5		
11	−5.8	4	2		−5.4	2	3	
12	−6.8	5	2	1	−6.7	6	3	

MAO‐B, as the most computationally studied protein of the two, was an ideal starting point for comparing the binding of these compounds. According to the docking scores for MAO‐B given in Table 1, SCRA compounds **1**–**5** have the strongest binding interaction with the protein, with 5F‐PB‐22 **3** and AM‐2201 **4** exhibiting the highest binding free energies of > 10.0 kcal·mol^−1^. Indeed, these results suggest that the addition of a group in the ‘head’ position of the SCRA structure increases the binding interaction with MAO‐B. This can be attributed to the larger size of the ligands, with greater potential for hydrophobic and hydrogen bonding interactions. Additionally, there will be limited availability of alternative configurations to fit in the binding pocket. If the ‘head’ group is an aromatic ring, as seen in both **3** and **4**, the ligand is also more rigid with fewer rotatable bonds, reducing the degrees of freedom and rendering the entropy less negative. Therefore, the ligand exhibits stronger binding to the protein. From Table 1, the data show that there is no significant difference in binding between compounds with indole or indazole as the ‘core’ group. This finding is logical given the structural similarities in compounds **1** and **2**, and **6**–**9**. The most common interacting residues in the active site of these calculations were consistent with previous literature; Leu 171, Gln 206, Tyr 326, Phe 343, Tyr 398 and Tyr 435 [38]. With tyrosine and phenylalanine both containing aromatic rings, the potential for π‐stacking interactions is high with **3** and **4**, although this has not been observed in any of the calculated docking poses in MAO‐B.

A remarkably similar pattern of binding interaction is observed with MAO‐A, with 5F‐PB‐22 **3** and AM‐2201 **4** remaining to be the strongest binding compounds. This is perhaps expected considering the structural similarity of both MAO proteins. The resulting π‐interaction between **3** and Tyr 407, as seen in Fig. 2, was the only parallel π‐stacking interaction identified in both proteins with all ligands, which will contribute to the increased binding interaction. Comparing compounds **6**–**9** to 1‐methyl‐1*H*‐Indole **10**, the binding scores are higher in both enzymes, indicating that the added intermolecular interactions between the hydrocarbon chain in the ‘tail’ position and the active site assist with stronger binding. The same pattern can also be observed for benzylamine **11**, which has the lowest binding free energies for both proteins, with the lowest number of interactions. The main residues involved with binding included Phe 208, Gln 215, Ile 335, Phe 352, and Tyr 407.

**Fig. 3:**
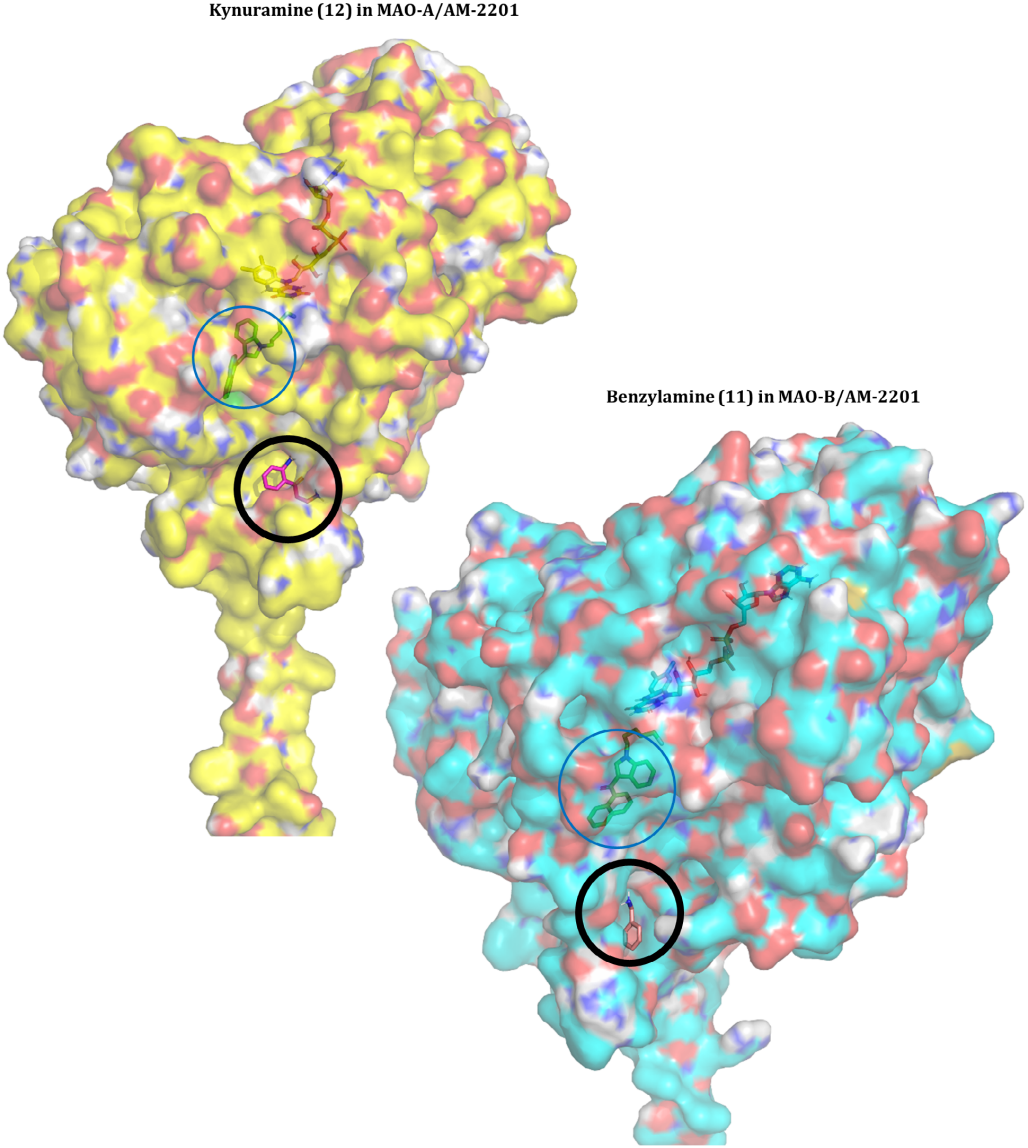
LEFT: Lowest energy binding pose of kynuramine (**12**, green) into the complex of AM‐2201 (**4**) in MAO‐A. RIGHT: Lowest energy binding pose of benzylamine (**11**, pink) into the complex of AM‐2201 (**4**) in MAO‐B. Compounds **11** and **12** can be seen on the outer surface of the MAO proteins, indicated by a black circle. AM‐2201 (**4**) is bound to the active site inside both proteins, indicated by a blue circle. The cofactor, FAD, can also be seen under the outer surface of the protein. Structure figures were generated using pymol (The PyMOL Molecular Graphics System, Version 2.4.1, Schrödinger, LLC).

To investigate the effect of inhibition from these ligands, the output file for the lowest energy binding pose of AM‐2201 **4** in both proteins was used for a further docking study. The docking of kynuramine **12** was attempted in the MAO‐A complex with AM‐2201 **4** and the docking of benzylamine **11** was attempted into the complex of MAO‐B and AM‐2201 **4**. The resultant configurations can be seen in Fig. 3. It is clear that **4** is large enough to take up available space in the active site and that this precludes access of the substrate to the FAD. That is, our docking studies suggest that SCRA binding is competitive with the substrate.

### Experimental kinetic inhibition studies

Given our *in‐silico* data suggests SCRAs might provide specific inhibition to MAO‐A and MAO‐B, we were encouraged to validate these data with experimental kinetic studies.

**Fig. 4:**
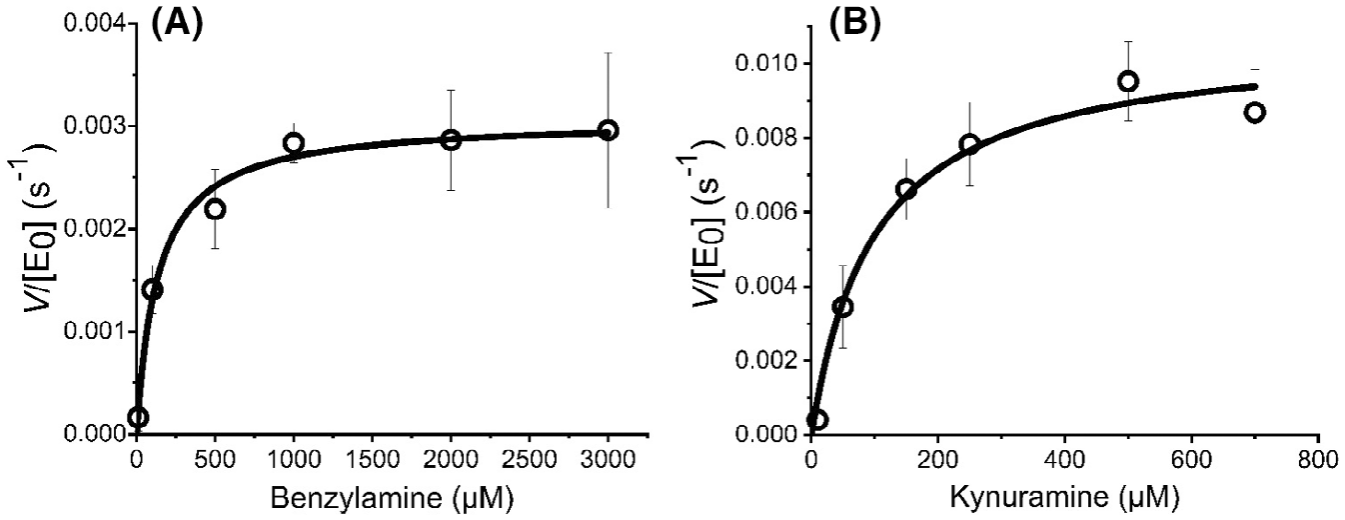
(A) Steady‐state kinetics plot of MAO‐B turnover, varying [Benzylamine **11**], *Conditions*, 30 μm MAO‐B, 50 mm HEPES, pH 7.5 + 0.5% Triton X‐100. (B) Steady‐state kinetics plot of MAO‐A turnover, varying [kynuramine **12**]. *Conditions*, 20 μm MAO‐A, 50 mm HEPES, pH 7.5 + 0.5% Triton X‐100 All data were recorded in triplicate and error bars represent the standard error.

First, we use MAO‐B as an exemplar system to study the molecular determinants of SCRA inhibition on MAO. At least in our hands, MAO‐B is more experimentally tractable with higher stability compared to MAO‐A and so we have focused the bulk of our analysis on this system. We have monitored the steady‐state kinetics of MAO‐B turnover using benzylamine **11** as the substrate and in the presence of increasing concentrations of each of **1**–**3** as shown in Fig. 1. Figure 4A shows example steady‐state turnover plots for MAO‐B that show a rectangular hyperbola, which can be adequately fit to the normal form of the Michaelis–Menten equation,

(1) $$v=\frac{V_{\max}\left[S\right]}{K_{M}+\left[S\right]}$$

giving *K*_M_ = 0.14 ± 0.03 mm.

**Fig. 5:**
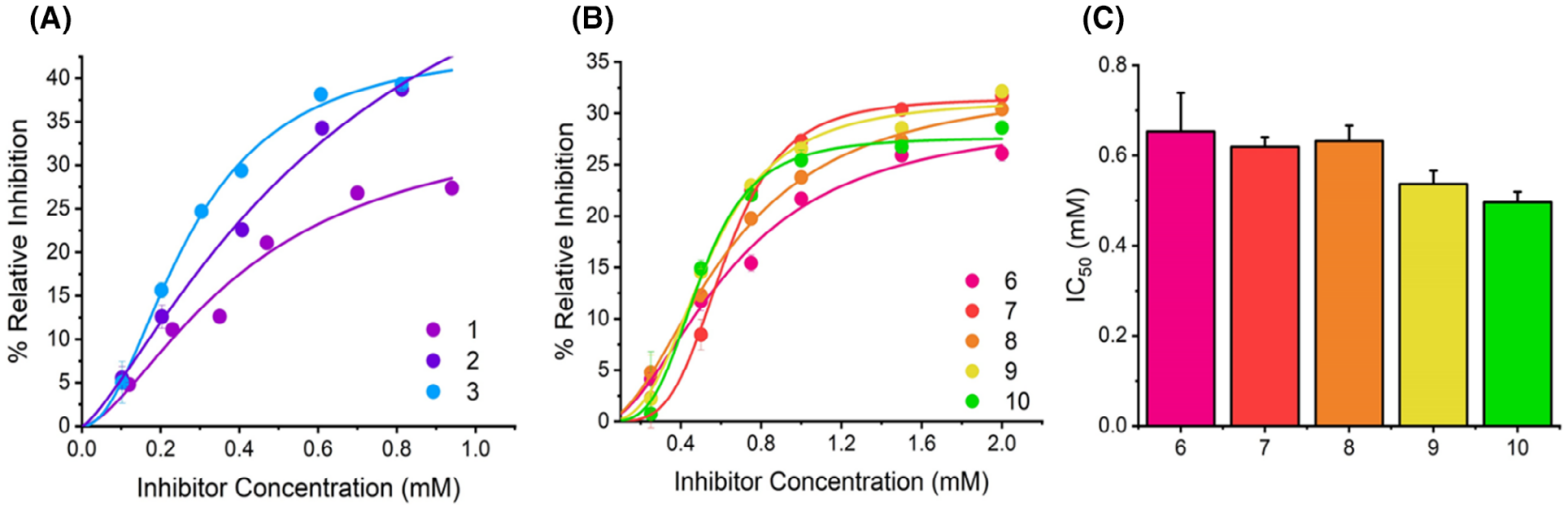
Concentration dependence of MAO‐B inhibition by SCRA compounds. (A) Concentration dependence of SCRA compounds **1**–**3** versus rate of MAO‐B turnover at 25 °C. Solid lines are the fit of the data to Eqn (2). (B) Concentration dependence of compounds **6**–**10** versus rate of MAO‐B turnover at 25 °C. Solid lines are the fit of the data to Eqn (2). (C) Resulting IC_50_ values depicting the inhibition potency of compounds **6**–**10**. *Conditions*, 30 μm MAO‐B, 1.5 mm BZA, 50 mm HEPES, pH 7.5 + 0.5% Triton X‐100, All data were collected in triplicate and error bars indicate standard error.

Figure 5 shows the concentration dependence of the inhibition by SCRAs, measured at saturating concentrations of substrate (> 10 X *K*_M_; 1.5 mm). In all cases a sigmodal relationship was found that could be fitted to the following equation:

(2) $$\%\text{Inhib}=\frac{I_{\max}{\left[I\right]}^{n}}{{{\mathrm{IC}}_{50}}^{n}+{\left[I\right]}^{n}\;}$$

where *n* indicates the level of divergence from a rectangular hyperbolic function and IC_50_ is the inhibition constant at 50% saturation of the inhibition percentage. For each SCRA, the data saturate below 100% inhibition, typically showing a maximal change in per cent inhibition of ~ 30%. The resulting IC_50_ and %max values are given in Table 2. We discuss the mechanistic interpretation of the inhibition data below.

**Table 2:** MAO *in vitro* inhibition resulting from the fit of Eqn (1) to the data shown in Fig. 5.

C1	MAO‐B			MAO‐A		
	Imax (%)	IC_50_ (mm)	*n*	Imax (%)	IC_50_ (μm)	*n*
1	36.45 ± 10.86	0.42 ± 0.19	1.59 ± 0.59	24.89 ± 0.83	87.82 ± 4.38	3.31 ± 0.46
2	70.81 ± 44.29	0.69 ± 0.42	1.30 ± 0.29	52.13 ± 1.50	77.10 ± 1.09	10.28 ± 2.19
3	44.29 ± 2.19	0.28 ± 0.02	2.03 ± 0.21	37.10 ± 0.36	19.32 ± 0.48	2.00 ± 0.09
5	NM	NM	NM	34.70 ± 8.17	49.15 ± 0.39	1.80 ± 0.64
6	29.94 ± 2.82	0.65 ± 0.09	1.94 ± 0.38	–	–	–
7	31.46 ± 0.95	0.62 ± 0.02	4.21 ± 0.55	–	–	–
8	33.10 ± 1.27	0.63 ± 0.03	1.98 ± 0.16	–	–	–
9	31.37 ± 1.27	0.54 ± 0.03	2.93 ± 0.45	–	–	–
10	26.68 ± 0.93	0.50 ± 0.02	3.72 ± 0.64	–	–	–

For the four SCRAs studied (**1**–**3**,**5**), we find a range of IC_50_ and *I*_max_ values. For **5**, we could not observe inhibition at technically accessible concentrations, given the solubility in MeOH. Structurally these SCRAs show individual unique differences, varying by either the core moiety (indole or indazole; **1** and **2**, respectively) or head group (tert‐leucinate or quinolynl group; **2** and **3**, respectively). Considering the trend in IC_50_ value, **3** has the smallest IC_50_ and **2** the largest. It is then tempting to speculate that the reason for the small IC_50_ value is the presence of the aromatic ring system at the head position, acknowledging that this also gives rise to an increase in *I*_max_, at least compared to **3** by ~ 15%. We note that the data in Fig. 5 suggest a very large potential *I*_max_ for **2**.

To study the molecular determinants of inhibition in more detail, we have synthesised the SCRA derivatives (**6**–**9**). When designing **6**–**9**, we focussed our study on the effect of (a) removing the head‐group, (b) the indole/indazole functional group and (c) modification/removal of the tail group. From Fig. 5B, **6**–**10** show sigmoidal character analogous to the original SCRA structures and have therefore been fit to Eqn (2). The parameters resulting from the fitting are given in Table 2.

From Table 2, we find that all the SCRA analogues (**6**–**9**) have similar *I*_max_ values and that these are also similar to 1‐methyl‐1*H*‐Indole (**10**). Both indazole derivatives, **7** and **9**, show a decrease in IC_50_ compared to their indole counterparts, **6** and **8**, but the difference is small and at least in the case of **6** and **7**, within the error of the measurement. We note that a decrease in IC_50_ for an indazole derivative is also evident for the SCRAs **1** and **2**, though again acknowledging the relatively large attendant error. Therefore, while there is a consistent trend for indazole analogues to have somewhat lower IC_50_ values, the difference would appear to be small. Clearer is the difference in the magnitude of *n*. From Table 2 we find an increase in *n* for the indazole derivatives (**7** and **9**) that is outside the error of the measurement. We discuss this difference in the context of the mechanism of inhibition below. There is no clear trend in any of the extracted parameters for variation in the tail group (at least fluorination).

Combined, our data suggest that an indazole core and an aromatic head group are determinants of low IC_50_ values for MAO‐B. However, the most significant determinant is the nature of the head group. The magnitude of the IC_50_ values is relatively large (hundreds of μm) and is a similar order magnitude for the SCRAs, the analogues and the simplest comparator, 1‐methyl‐1*H*‐Indole (**10**).

Having established that SCRAS act as MAO‐B inhibitors, we then turned our study to MAO‐A, the alternative monoamine isoform. The monoamine oxidases share similar structures (70% sequence identity), molecular weights and each have hydrophobic active sites [23, 24, 34]. In general, the MAO isoforms show differing selectivity for substrates and inhibitors [24, 25, 26, 27, 28, 29, 30, 31, 32, 33]. Given our data for MAO‐B showed the key determinant for lowering IC_50_ arose from the nature of the head group, we have selected 4 molecules from Fig. 1 to track variation in hydrophobicity and bulk, namely **1** (indazole; tert‐leucinate), **2** (indole; tert‐leucinate), **3** (quinolynl) and **5** (iodobenzene). From Table 1, these molecules are calculated to have a progressive increase in docking score (3 > 5 > 1 and 2; −10.6, −9.4, −9.0 kcal·mol^−1^).

**Fig. 6:**
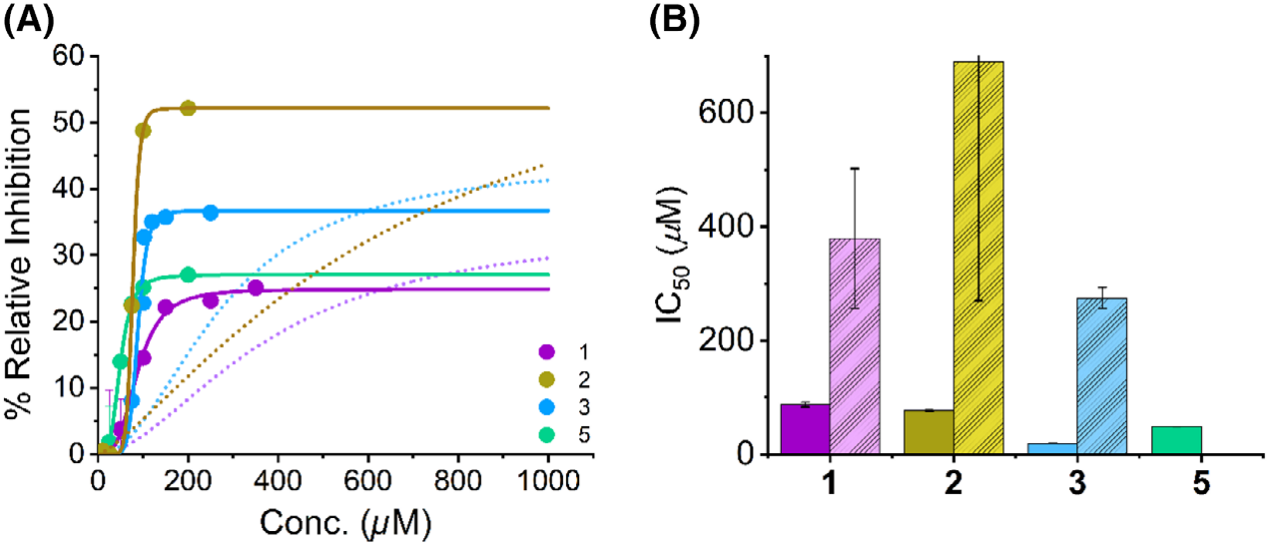
Concentration dependence of MAO‐A inhibition by SCRA compounds. MAO‐A turnover in the presence of three SCRA compounds. (A) Concentration dependence of SCRA compounds **1**,**3**,**5** versus rate of MAO‐A turnover at 25 °C. Solid lines are the fit of the data to Eqn (2), dashed lines are fit of corresponding MAO‐B data. (B) Resulting IC_50_ values depicting the inhibition potency of SCRA compounds **1**,**3**,**5**, with the corresponding IC_50_ values for MAO‐B shown in pastel. *Conditions*, 20 μm MAO‐A, 1 mm KYN, 50 mm HEPES, pH 7.5 + 0.5% Triton X‐100, All data were collected in triplicate and error bars indicate standard error.

We performed analogous inhibition kinetics experiments using kynuramine **12** as the substrate (Fig. 4B), giving *K*_M_ = 0.14 ± 0.03 mm. This allowed us to compare the potency of inhibition between the two MAO isoforms. The resulting inhibition plots are shown in Fig. 6. As with MAO‐B, the data show inhibition saturation with a sigmoidal like relationship to SCRA concentration. We have therefore fit the data using Eqn (2) and the resulting data from the fitting is given in Table 2. From Fig. 6 and Table 2 the range of *I*_max_ values is similar to MAO‐B, with average and standard deviation; 32.2 ± 6.5% for MAO‐A *versus* 50.5 ± 18% for MAO‐B. The extracted IC_50_ values directly mirror the trend in the calculated affinities; **3** is the most potent (19.3 ± 0.5 μm) and **1** the least potent inhibitor (87.8 ± 4.4 μm). Moreover, **1** also the smallest *I*_max_ being 24.9 ± 0.8% compared to 37.1 ± 0.4% for **3**. That is, the difference between a tert‐leucinate and quinolynl head group is sufficient to increase the inhibitor potency by ~ 5‐fold.

Compared to MAO‐B, the IC_50_ values are approximately an order magnitude smaller for MAO‐A, with average and standard deviation; 52 ± 34 μm for MAO‐A *versus* 460 ± 280 μm for MAO‐B. Specifically, 5F‐PB‐22 **3** and 5F‐ADB **1** show increases of ~ 14 fold and ~ 9 fold respectively between the MAO isoforms. From these data we can infer that SCRAs are MAO‐A selective inhibitors. Our data suggest a range of potencies of inhibitor depending on the specific SCRA head group, with increasingly hydrophobic, bulky groups being correlated with a smaller IC_50_.

Our docking studies provide a means to interpret the experimentally observed selectivity of MAO‐A for certain SCRA analogues vs. MAO‐B. From Fig. 2, MAO‐B has a smaller, more restrictive entrance to its active site, which we suggest impedes the binding of larger head groups [24, 33, 34]. These binding characteristics have been successfully employed in targeted design of MAO inhibitor molecules [30, 45, 46, 47, 48].

### Mechanism of SCRA MAO inhibition

**Fig. 7:**
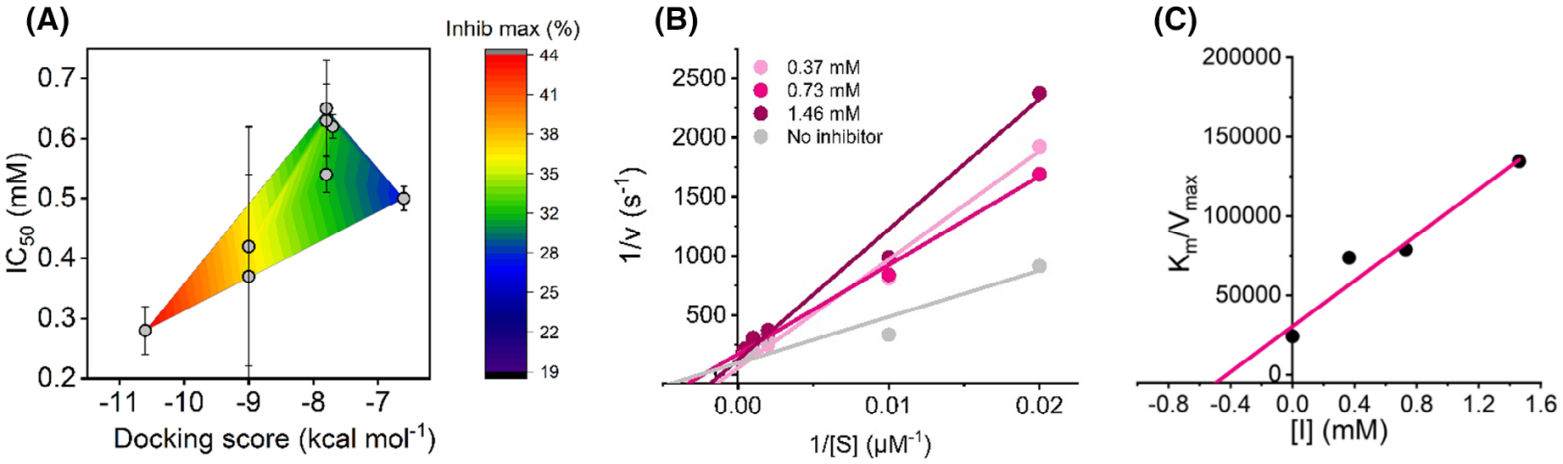
Additional kinetic investigation into the SCRA inhibition of MAO‐B turnover. (A) Comparison of *in silico* and *in vitro* data; the experimentally determined IC_50_ values of eight compounds are plotted against the computationally determined docking score. The overlaid heat map indicates the relationship of the maximum % inhibition with respect to the other parameters. (B) Kinetics study of the mechanism of MAO‐B inhibition by compound **8**. A Lineweaver‐Burk plot for MAO‐B inhibition by **8** has been plotted where substrate concentrations of 50–3000 mm BZA were used in conjunction with three inhibitor concentrations. (C) Plot of *K*_M_/*V*_max_ versus inhibitor concentration for the determination of the *K*_i_ value of compound 8. *Conditions*, 24 μm MAO‐B, 50 mm HEPES, pH 7.5 + 0.5% Triton X‐100, All data were collected in triplicate and error bars indicate standard error.

Figure 7A shows the correlation between our experimentally extracted inhibition data and the docking scores from our docking studies (Fig. 2). From Fig. 7A there is an evident positive correlation between the docking scores and the extracted IC_50_ and *I*_max_ values: The data have a calculated Pearson coefficient of 0.56. However, we note the large error for some of the values. However, we note the large error for some of the values. The direct correlation with experiment suggests the binding geometries identified from our docking studies are accurate. From Fig. 2 (as we describe above), these studies suggest SCRA binding may be competitive with the normal substrate and that, without conformational change, SCRA and substrate binding would be mutually exclusive. Figure 7B,C show the concentration dependence of **8** on MAO‐B turnover. These data show an increase in the apparent *K*_M_ value with increasing concentration of **8**. This finding is a classical kinetic relationship that characterises competitive inhibition and tracks directly with the findings of our docking studies. While the SCRA binding precludes access to the flavin in our docking studies (Fig. 3), it would be interesting to understand the structural relationship over time, not least because we have recently shown that MAO‐B motions during turnover are important [49].

The observation of a sigmoidal relationship with respect to inhibitor concentration (Figs 5 and 6) and at saturating substrate concentrations is suggestive of an allosteric model of inhibition. The magnitude of *n* characterises the sensitivity of the allosteric effect. From Table 2, we find the value is in the range *n* ≈ 1.5–10. There is no obvious trend in the magnitude of *n* and either IC_50_ or *I*_max_ values. Sigmoidal plots of per cent inhibition at saturating substrate concentrations arise where the inhibitor preferentially binds to an inactive/less active form of the enzyme. The observation of < 100% inhibition is consistent with the notion of the SCRA‐bound enzyme having a decreased, but not zero, rate of turnover. That is, increasing saturation of the inhibitor‐bound form will result in a less active, but not inactive enzyme.

Together our data suggest competitive, allosteric inhibition, which drives the formation of a less active enzyme. The simplest mechanistic model is then one where SCRA binding includes a conformational change, allowing substrate binding but in a less optimal geometry, giving rise to a decrease in the observed rate of turnover.

## Conclusions

Combined, our computational and experimental data show that SCRAs can act as MAO‐A selective inhibitors and that the nature of the SCRA head group is a key determinant in the affinity of the SCRA. In particular, we note that the π‐interaction between the SCRA and Tyr 407 in MAO‐A appears to be a key determinant of this specificity/affinity. Our data suggest the mode of inhibition maybe complex, likely involving a competitive allosteric effect, which decreases the rate of MAO turnover.

The use of MAO‐I's has long been associated with the potential for serious cardiovascular events when accompanied by the ingestion of high levels of dietary tyramine, known as the tyramine pressor response [27, 37]. Tyramine, a biogenic amine, is commonly found in certain food types and dietary control is required to reduce the risk of hypertensive crisis upon the administration of MAO‐I to patients [27]. Tyramine consumption causes an increase in blood pressure or ‘pressor response’, however under normal conditions this effect is negligible as Tyr is easily oxidised by the MAO enzymes. When combined with the use of MAO‐I, the level of Tyr reaching the systemic circulation is much higher due to the absence of first‐pass metabolism of Tyr by MAOIs in the liver. This causes various effects, such as the release of high levels of adrenaline and noradrenaline, which lead to adrenergic toxicity and hypertensive events [27, 29, 34].

These interactions have been well studied and despite both MAO isoforms showing similar affinities for tyramine, the pressor response has been primarily linked to the selective inhibition of MAO‐A [29]. This is due to the predominance of this monoamine isoform in the intestine and liver, and the greater affinity MAO‐A has for adrenaline and noradrenaline than MAO‐B [37]. Substantial pressor effects can be provoked by the excessive consumption of Tyr‐rich foods on an empty stomach [27]. For example, beer is Tyr‐rich liquid, with the average European beverage containing 7 mg·L^−1^ Tyr, which when drunk in moderation (two servings; 500 mL) would not pose a significant Tyr pressor response. However, it is important to consider when consumed in excess and on an empty stomach, concentrations could become very high and this scenario is likely in particular in the homeless community, where there can be very high rates of SCRA use. Given our data show SCRAs are MAO‐A selective inhibitors, a tyramine pressor response, precipitated by smoking certain SCRAs, could therefore provide an explanation for the severe and unpredictable hypertensive side effects recorded in the using community.

**Fig. 8:**
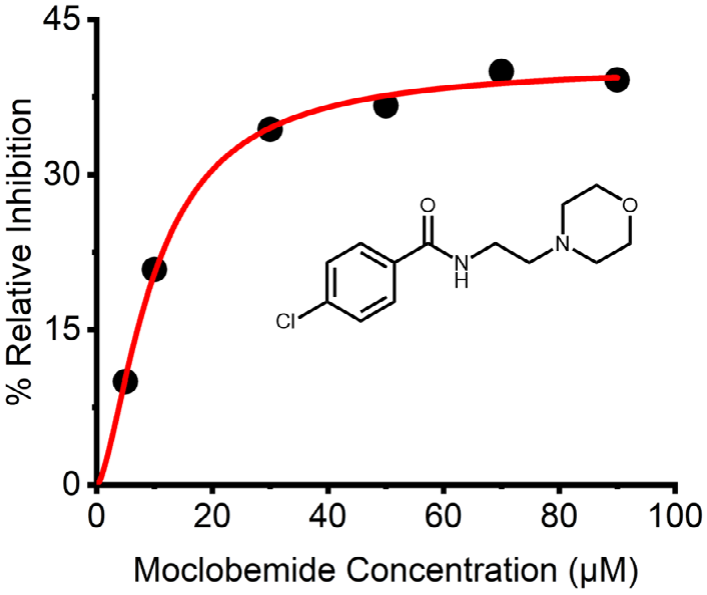
Concentration dependence of MAO‐A inhibition by known inhibitor Moclobemide (Structure known above). Concentration dependence of Moclobemide versus rate of MAO‐A turnover at 25 °C. Solid lines are the fit of the data to Eqn (2). *Conditions*, 20 μm MAO‐A, 1 mm KYN, 50 mm HEPES, pH 7.5 + 0.5% Triton X‐100, All data were collected in triplicate.

We acknowledge the extracted IC_50_ values are in the micromolar range, which is rather larger than for clinically used MAO‐Is. For example, the potent MAO‐A inhibitors, Clorgyline and *β*‐carboline harmaline exhibit IC_50_'s of 16 and 20 nm respectively. However, this is not the case for all established inhibitors. For example, Toloxatone and Moclobemide, both MAO‐A selective antidepressant drugs, have reported IC_50_'s of 6.71 and 500 μm respectively. Indeed, when tested with the assay used in this study, we find an IC50 of 9.94 μm for Moclobemide (Fig. 8). Both compounds are considered potent MAO‐Is despite their IC_50_'s, due to the metabolites they form *in vitro*. As such, the study of SCRA's *in vivo* should be considered.

Having established the IC_50_ values of these SCRA compounds *in vitro*, it is important to consider these values in a clinically relevant context. When studying the ACMD SCRA report alongside a conciliated database of quantified post‐mortem toxicology reports, it is postulated the average concentration of SCRA in the blood falls between 1.32 and 6.62 nm with values up to 0.5 μm observed. Moreover, it has been suggested that such compounds are unstable *in vivo* and post‐pyrolysis and as such this must be factored into any concentrations reported.

We note that the enzyme system is in a non‐native environment (detergent rather than the mitochondrial membrane) and we have recently demonstrated that differences in the lipid environment affect MAO‐B activity [49]. Our data therefore provide the rationale for the need to study the effect of SCRA‐mediated MAO inhibition in an *in vivo* model, to establish further the rationale for harm reduction advice associated with SCRA use and the potential for negative side‐effects associated with the tyramine pressor response.

## Materials and methods

### Kinetic measurements

All reactants were pre‐incubated at 25 °C in 50 mm HEPES (pH 7.5), containing 0.5% (w/v) reduced Triton X‐100. Kinetics data were collected using a UV/Vis spectrophotometer (Agilent Cary 60 UV‐Vis spectrometer) fitted with temperature regulation, in 3 mm quartz cuvette. For MAO‐B kinetic data, reactions were initiated by the addition of MAO‐B and the formation of benzaldehyde was monitored using ε_250_ = 12 800 m ^−1^·cm^−1^ [50]. For MAO‐A kinetic data, reactions were initiated by the addition of MAO‐A and the formation of 4‐hydroxyquinoline was monitored using ε_316_ = 12 300 m ^−1^·cm^−1^ [51]. The data were collected in triplicate and with all steady‐state kinetics fitting well to the Michaelis–Menten equation (Fig. 4). Initial rates were typically collected over 2 min.

### MAO inhibition kinetics

All inhibitory kinetics measurements were performed using the conditions stated above. For MAO‐B inhibition by compounds **1**–**3** and **6**–**10**, 1.5 mm benzylamine **11** (10 × *K*_M_) was used in conjunction with 30 μm of enzyme. For MAO‐A inhibition by compounds **1**,**3**,**5**, 1 mm kynuramine **12** (10 × *K*_M_) was used in conjunction with 20 μm of enzyme. In all cases, the inhibitory SCRA compounds were dissolved in MeOH, therefore MeOH controls were recorded, where MeOH concentration was kept as low as possible and did not exceed 10% of the assay volume. All conditions were measured in triplicate.

### Compounds

Synthetic cannabinoid reference materials of 5F‐ADB **1**, 5F‐MDMB‐PICA **2**, 5F‐PB‐22 **3**, and AM‐694 **5**, were purchased from Cayman Chemical (Cambridge, UK). All other compounds were purchased from Merck (Gillingham, UK).

### Synthesis of compound **6** & **7**

Under N_2_, sodium hydride (60% in mineral oil, 0.3942 g, 9.860 mmol) was dissolved in DMF (16.4 mL) and stirred at 0 °C. Indole or indazole (4.930 mmol) in DMF (5.0 mL) was added to the sodium hydride solution at 0 °C and stirred for 30 min. A solution of 1‐bromo‐5‐fluoropentane (5.916 mmol, 1.000 g) in DMF (5 mL) was added to the mixture at 0 °C, allowed to gradually heat to room temperature and stirred for 14 h (overnight). Methanol (6 mL) and water (9 mL) were added at 0 °C to quench the reaction, and the compound was extracted with DCM (3 × 20 mL), water (2 × 30 mL) and 1 m sodium chloride solution (1 × 30 mL). After drying over MgSO_4_, all solvent was removed in vacuo. The crude product was then purified by column chromatography (pentane: ethyl acetate).

### 1‐(5‐fluoropentyl)‐1H‐Indole **6**

Pale‐yellow oil (0.4167 g, 2.03 mmol, 41.2%); ^1^H NMR (500 MHz, CDCl_3_) δ 7.64 (dt, J = 7.9, 0.9 Hz, 1H), 7.34 (dt, J = 8.2, 0.9 Hz, 1H), 7.21 (ddd, J = 8.1, 7.0, 1.1 Hz, 1H), 7.13–7.07 (m, 2H), 6.49 (dd, J = 3.1, 0.8 Hz, 1H), 4.46 (t, J = 6.0 Hz, 1H), 4.37 (t, J = 6.0 Hz, 1H), 4.15 (t, J = 7.0 Hz, 2H), 1.90 (dt, J = 15.0, 7.2 Hz, 2H), 1.78–1.64 (m, 2H), 1.50–1.40 (m, 2H) ppm; ^13^C NMR (126 MHz, CDCl_3_) δ 136.05, 128.75, 127.87, 121.53, 121.13, 119.38, 109.43, 101.19, 84.57, 83.26, 46.40, 30.26, 30.10, 30.06, 23.04, 23.00 ppm; ^19^F NMR (376 MHz, CDCl_3_) δ −218.51 (s, 1F); IR (ATR) 3051.45 (Ar‐H), 2940.68 (Ar‐H), 2868.21 (Ar‐H), 1463.12 cm^−1^; *m*/*z*: [M + H]^+^ Calculated for C_13_H_16_NF 205.1267; Found 205.1271.

### 1‐(5‐fluoropentyl)‐1H‐Indazole **7**

Pale‐yellow oil (0.3544 g, 1.78 mmol, 34.9%); ^1^H NMR (500 MHz, CDCl_3_) δ 7.99 (d, J = 0.9 Hz, 1H), 7.73 (dt, J = 8.1, 1.0 Hz, 1H), 7.43–7.35 (m, 2H), 7.14 (ddd, J = 7.9, 6.5, 1.2 Hz, 1H), 4.48–4.33 (m, 4H), 1.99 (dt, J = 15.0, 7.2 Hz, 2H), 1.79–1.66 (m, 2H), 1.44 (tt, J = 10.1, 6.5 Hz, 2H); ^13^C NMR (126 MHz, CDCl_3_) δ 139.54, 132.95, 126.30, 124.14, 121.30, 120.57, 109.03, 84.57, 83.26, 77.41, 77.16, 76.91, 48.78, 30.21, 30.05, 29.60, 22.86, 22.82. ^19^F NMR (376 MHz, CDCl_3_) δ −218.48 (s, 1F); IR (ATR) 3059.11 (Ar‐H), 2938.76 (Ar‐H), 2866.72 (Ar‐H), 1615.57 (Ar‐C=C), 1498.98, 1465.00 cm^−1^; *m*/*z*: [M + H]^+^ Calculated for C_12_H_15_N_2_F 206.1219; Found 206.1222.

### Synthesis of compounds **8** & **9**

Under N_2_, sodium hydride (60% in mineral oil, 0.497 g, 12.43 mmol) was dissolved in DMF (20.7 mL) and stirred at 0 °C. Indole or indazole (11.3 mmol) in DMF (11.3 mL) was added to the sodium hydride solution at 0 °C and stirred for 30 min. A solution of 1‐bromopentane (16.95 mmol, 2.5601 g, 2.1 mL) in DMF (5.7 mL) was added to the mixture at 0 °C, allowed to gradually heat to room temperature and stirred for 1 h. Water (20 mL) was added at 0 °C to quench the reaction, and the compound was extracted with EtOAc (3 × 25 mL) and water (2 × 30 mL). After drying over MgSO_4_, all solvent was removed in vacuo. The crude product was then purified by column chromatography (Pentane: Ethyl Acetate).

### 1‐pentyl‐1H‐Indole **8**

Yellow oil (0.3986 g, 2.13 mmol, 18.8%); ^1^H NMR (126 MHz, CDCl_3_) δ 7.63 (d, J = 7.9 Hz, 1H), 7.35 (d, J = 8.3 Hz, 1H), 7.20 (ddd, J = 8.3, 7.0, 1.2 Hz, 1H), 7.13–7.06 (m, 2H), 6.49 (dd, J = 3.1, 0.9 Hz, 1H), 4.12 (t, J = 7.1 Hz, 2H), 1.85 (p, J = 7.2 Hz, 2H), 1.41–1.24 (m, 4H), 0.89 (t, J = 6.9 Hz, 3H) ppm; ^13^C NMR (500 MHz, CDCl_3_) δ 136.10, 128.71, 127.91, 121.41, 121.06, 119.27, 109.51, 100.95, 77.41, 77.16, 76.91, 46.55, 30.11, 29.31, 22.49, 14.10 ppm; IR (ATR) 3054.84 (Ar‐H), 2955.23 (Ar‐H), 2929.44 (Ar‐H), 2871.24 (Ar‐H), 1463.11 cm^−1^; *m*/*z*: [M + H]^+^ Calculated for C_13_H_17_N 187.1361; Found 187.1365.

### 1‐pentyl‐1H‐Indazole **9**

Yellow oil (1.2228 g, 6.50 mmol, 57.5%); ^1^H NMR (500 MHz, CDCl_3_) δ 7.99 (d, J = 0.9 Hz, 1H), 7.73 (dt, J = 8.1, 1.0 Hz, 1H), 7.43–7.34 (m, 2H), 7.13 (ddd, J = 7.9, 6.7, 1.1 Hz, 1H), 4.38 (t, J = 7.2 Hz, 2H), 1.93 (p, J = 7.3 Hz, 2H), 1.40–1.26 (m, 4H), 0.88 (t, J = 7.0 Hz, 3H); ^13^C NMR (126 MHz, CDCl_3_) δ 139.51, 132.78, 126.16, 124.11, 121.25, 120.46, 109.14, 77.41, 77.16, 76.91, 49.07, 29.72, 29.18, 22.46, 14.08. IR (ATR) 3062.59 (Ar‐H), 2956.37 (Ar‐H), 2931.05 (Ar‐H), 2859.84 (Ar‐H), 1615.71 (Ar‐C=C), 1498.91, 1464.94 cm^−1^; *m*/*z*: [M + H]^+^ Calculated for C_12_H_16_N_2_ 188.1313; Found 188.1316.

### Flexible docking in Autodock

3D crystal structures were downloaded from RSCB Protein Data Bank for both MAO‐A (PDB: 2Z5X) and MaAO‐B (PDB: 2V5Z). Both crystal structures were prepared in autodocktools 1.5.6. To prepare the proteins, the bound inhibitors were removed alongside all water molecules and any heteroatoms apart from FAD. Polar hydrogens were added and Kollman charges were calculated. Flexible docking was achieved by setting flexible residues for each protein in close proximity to the active site. The side chain residues Ile 180, Gln 215, Ile 335, Leu 337, Phe 352, Tyr 407, and Tyr 444 were chosen as flexible residues for Mao‐A, and residues Leu 171, Ile 199, Tyr 326, Phe 343, and Tyr 398 in Mao‐B. All other residues remained rigid and all rotatable bonds could freely rotate. The ligand chemical structures were drawn on chem3d 16.0 software and the energy was initially minimised using the MM2 force field. All structures were further optimised using DFT, with geometry optimisations being performed in Gaussian 16 (Rev. A.03). Calculations were completed at the B3LYP/6‐31 g level of theory to find the geometry of the compounds at their energy minima. Flexible docking was then undertaken using autodock vina by selecting certain residues in the protein active site and labelling them as flexible. All other residues remained rigid. All nine output configurations were inspected for location in protein and interactions with residues. The lowest energy conformation for each compound was used for comparison. Interactions were further investigated using the Protein‐Ligand Interaction Profiler [37].

## Conflict of interest

The authors declare no conflict of interest.

## Author contributions

SAH, RCA, CRP – Planned experiments, performed experiments, analysed data, contributed reagents, wrote the paper. MJD, MWvdK, AEM, OS, TFH, TF, JS, SH, IB, JLRA, DC – Planned experiments, Guidance given on experiments, Edited Paper.

### Peer review

The peer review history for this article is available at https://publons.com/publon/10.1111/febs.16741.

## Supporting information


**Fig. S1.** Validation study to compare Autodock 4.2 method with the co‐crystallised inhibitors within MAO‐A (left) and MAO‐B (right).
**Fig. S2.** Lowest energy binding poses between ligands and residues in the active site of MAO‐A.
**Fig. S3.** Lowest energy binding poses between ligands and residues in the active site of MAO‐B.
**Fig. S4.** 1H NMR for N‐5‐fluoropentylindole, 6.
**Fig. S5.** 13C NMR for N‐5‐fluoropentylindole, 6.
**Fig. S6.** 19F NMR for N‐5‐fluoropentylindole, 6.
**Fig. S7.** IR spectrum for N‐5‐fluoropentylindole, 6.
**Fig. S8.** MS confirmation for N‐5‐fluoropentylindole, 6.
**Fig. S9.** 1H NMR for N‐5‐fluoropentylindazole, 7.
**Fig. S10.** 13C NMR for N‐5‐fluoropentylindazole, 7.
**Fig. S11.** 19F NMR for N‐5‐fluoropentylindazole, 7.
**Fig. S12.** IR spectrum for N‐5‐fluoropentylindazole, 7.
**Fig. S13.** MS confirmation for N‐5‐fluoropentylindazole, 7.
**Fig. S14.** 1H NMR for N‐pentylindole, 8.
**Fig. S15.** 13C NMR for N‐pentylindole, 8.
**Fig. S16.** IR spectrum for N‐pentylindole, 8.
**Fig. S17.** MS confirmation for N‐pentylindole, 8.
**Fig. S18.** 1H NMR for N‐pentylindazole, 9.
**Fig. S19.** 13C NMR for N‐pentylindazole, 9.
**Fig. S20.** IR spectrum for N‐pentylindazole, 9.
**Fig. S21.** MS confirmation for N‐pentylindazole, 9.

## Data Availability

The authors confirm that the data supporting the findings of this study are available within the article and its supplementary materials.
