# Supplementary material for: Synthetic cannabinoid receptor agonists are monoamine oxidase‐A selective inhibitors
Source: FEBS J. 2023 Feb 21;290(12):3243–57. doi: 10.1111/febs.16741 (PMC10952593; doi:10.1111/febs.16741)
Supplement: Supplementary file 1 — Fig. S1. Validation study to compare Autodock 4.2 method with the co‐crystallised inhibitors within MAO‐A (left) and MAO‐B (right). Fig. S2. Lowest energy binding poses between ligands and residues in the active site of MAO‐A. Fig. S3. Lowest energy binding poses between ligands and residues in the active site of MAO‐B. Fig. S4. 1H NMR for N‐5‐fluoropentylindole, 6. Fig. S5. 13C NMR for N‐5‐fluoropentylindole, 6. Fig. S6. 19F NMR for N‐5‐fluoropentylindole, 6. Fig. S7. IR spectrum for N‐5‐fluoropentylindole, 6. Fig. S8. MS confirmation for N‐5‐fluoropentylindole, 6. Fig. S9. 1H NMR for N‐5‐fluoropentylindazole, 7. Fig. S10. 13C NMR for N‐5‐fluoropentylindazole, 7. Fig. S11. 19F NMR for N‐5‐fluoropentylindazole, 7. Fig. S12. IR spectrum for N‐5‐fluoropentylindazole, 7. Fig. S13. MS confirmation for N‐5‐fluoropentylindazole, 7. Fig. S14. 1H NMR for N‐pentylindole, 8. Fig. S15. 13C NMR for N‐pentylindole, 8. Fig. S16. IR spectrum for N‐pentylindole, 8. Fig. S17. MS confirmation for N‐pentylindole, 8. Fig. S18. 1H NMR for N‐pentylindazole, 9. Fig. S19. 13C NMR for N‐pentylindazole, 9. Fig. S20. IR spectrum for N‐pentylindazole, 9. Fig. S21. MS confirmation for N‐pentylindazole, 9. [file FEBS-290-3243-s001.docx]

*Supporting Information*: Synthetic Cannabinoid Receptor Agonists are Monoamine Oxidase-A Selective Inhibitors

Sarah A Hindson ,^1‡^ Rachael C Andrews ,^2,3‡^ Michael J Danson ,^1^ Marc W van der Kamp,^4^ Amy E Manley,^5^ Oliver Sutcliffe,^6^ Thomas Fincham-Haines,^7^ Thomas Freeman,^8^ Jennifer Scott,^5^ Stephen Husbands,^9^ Ian Blagbrough,^9^ JL Ross Anderson,^4^ David Carbery,^2,3^ Christopher R Pudney^1,3,10^*

^1^Department of Biology and Biochemistry, ^2^Department of Chemistry, ^3^Centre for Sustainable and Circular Technologies, University of Bath, Bath BA2 7AY, UK. ^4^School of Biochemistry, University of Bristol, Bristol, BS8 1TD. ^5^Faculty of Health Sciences, University of Bristol, Bristol, BS8 1TH. ^6^MANchester DRug Analysis & Knowledge Exchange (MANDRAKE), Department of Natural Sciences, Manchester Metropolitan University, Manchester, M15 5GD, UK. ^7^Department of Computer Science, ^8^Department of Psychology, ^9^Department of Pharmacy and Pharmacology, Centre for Therapeutic Innovation, University of Bath, Bath, BA2 7AY, UK.

**Contents:**

**Figure S1**. Validation study to compare Autodock 4.2 method with the co-crystallized inhibitors within MAO-A (left) and MAO-B (right).

**Figure S2.** Lowest energy binding poses between ligands and residues in the active site of MAO-A.

**Figure S3.** Lowest energy binding poses between ligands and residues in the active site of MAO-B.

**Figure S4.** ^1^H NMR for N-5-fluoropentylindole, 6

**Figure S5.** ^13^C NMR for N-5-fluoropentylindole, 6.

**Figure S6.** ^19^F NMR for N-5-fluoropentylindole, 6.

**Figure S7.** IR spectrum for N-5-fluoropentylindole, 6.

**Figure S8.** MS confirmation for N-5-fluoropentylindole, 6.

**Figure S9.** ^1^H NMR for N-5-fluoropentylindazole, 7.

**Figure S10.** ^13^C NMR for N-5-fluoropentylindazole, 7.

**Figure S11.** ^19^F NMR for N-5-fluoropentylindazole, 7.

**Figure S12.** IR spectrum for N-5-fluoropentylindazole, 7.

**Figure S13.** MS confirmation for N-5-fluoropentylindazole, 7.

**Figure S14.** ^1^H NMR for N-pentylindole, 8.

**Figure S15.** ^13^C NMR for N-pentylindole, 8.

**Figure S16.** IR spectrum for N-pentylindole, 8.

**Figure S17.** MS confirmation for N-pentylindole, 8.

**Figure S18.** ^1^H NMR for N-pentylindazole, 9.

**Figure S19.** ^13^C NMR for N-pentylindazole, 9.

**Figure S20.** IR spectrum for N-pentylindazole, 9.

**Figure S21.** MS confirmation for N-pentylindazole, 9.

**Figures**


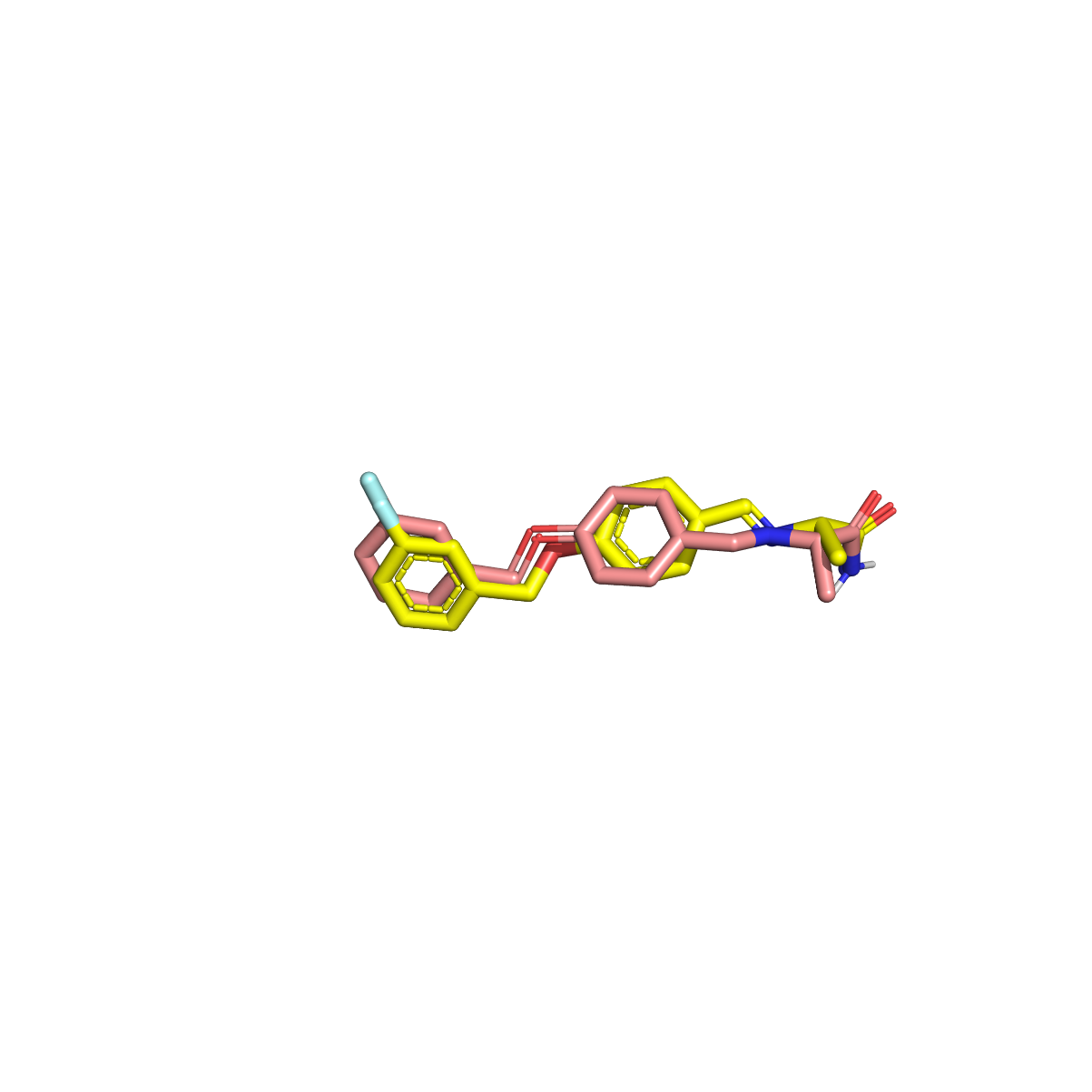

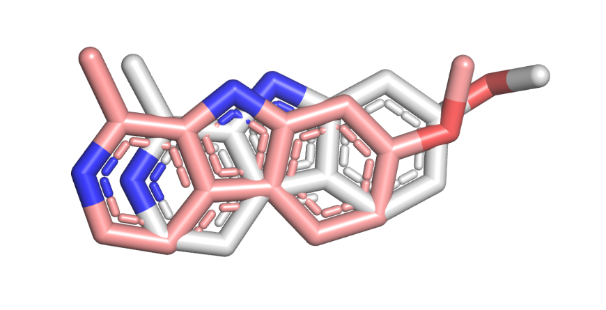


**Figure S1**. Validation study to compare Autodock 4.2 method with the co-crystallized inhibitors within MAO-A (left) and MAO-B (right). Following removal of co-crystalised inhibitor from the original pdb files, these compounds were then used for in-silico binding via Autodock Vina to validate the method in use during this study. The RMSD of the lowest energy binding pose and the co-crystalised configuration was compared. In both examples, the pink-coloured compound is the co-crystalised geometry obtained from the original pdb file. The RMSD of harmine in MAO-A (left) is 1.237 Å and RMSD of safinamide in MAO-B (right) is 0.965 Å, calculated using DockRMSD software.^42^ These RMSD values are below the accepted value of 2.0 Å for RMSD scoring.^38^ Structure figures were generated using PyMol (The PyMOL Molecular Graphics System, Version 2.4.1, Schrödinger, LLC).


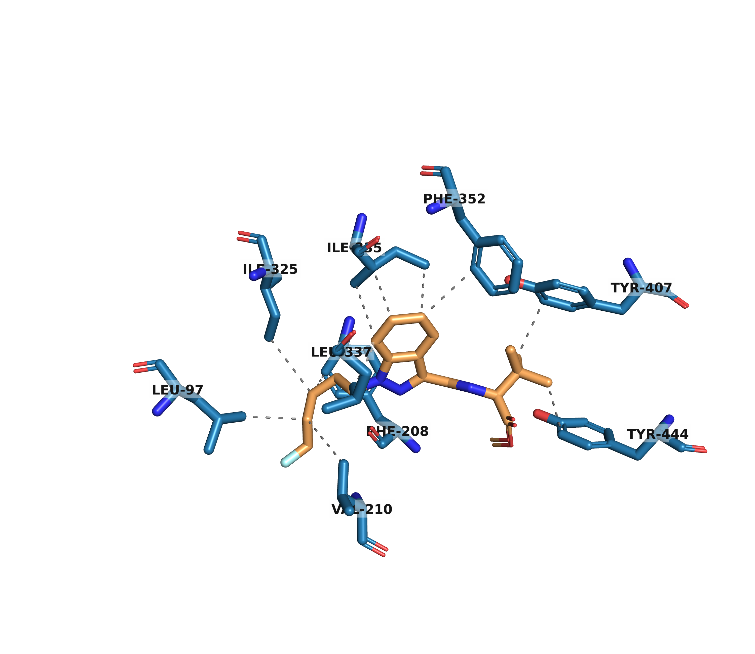


**5F-ADB (1) in MAO-A**


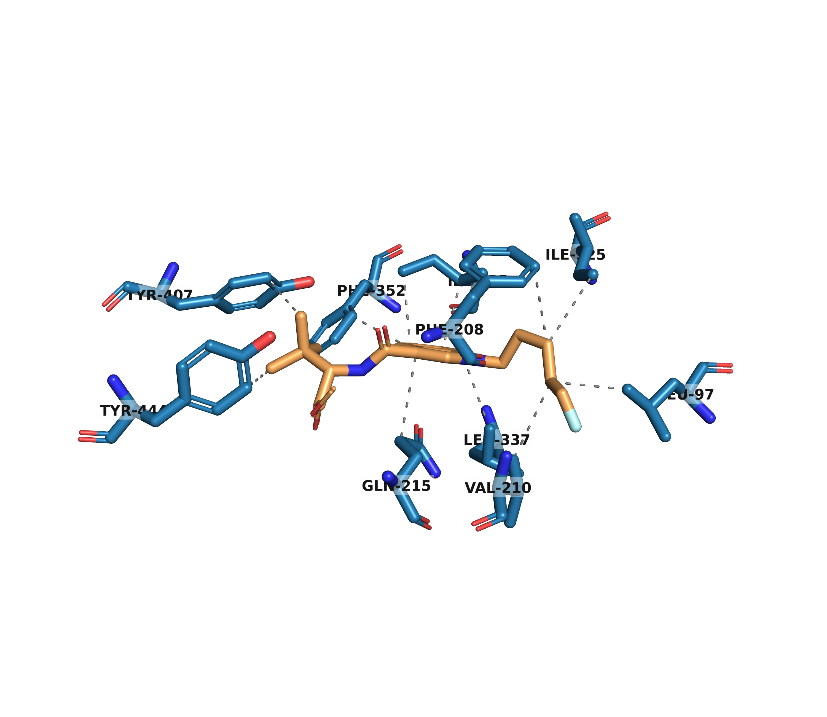


**5F-MDMB-PICA (2) in MAO-A**

**AM-2201 (4) in MAO-A**


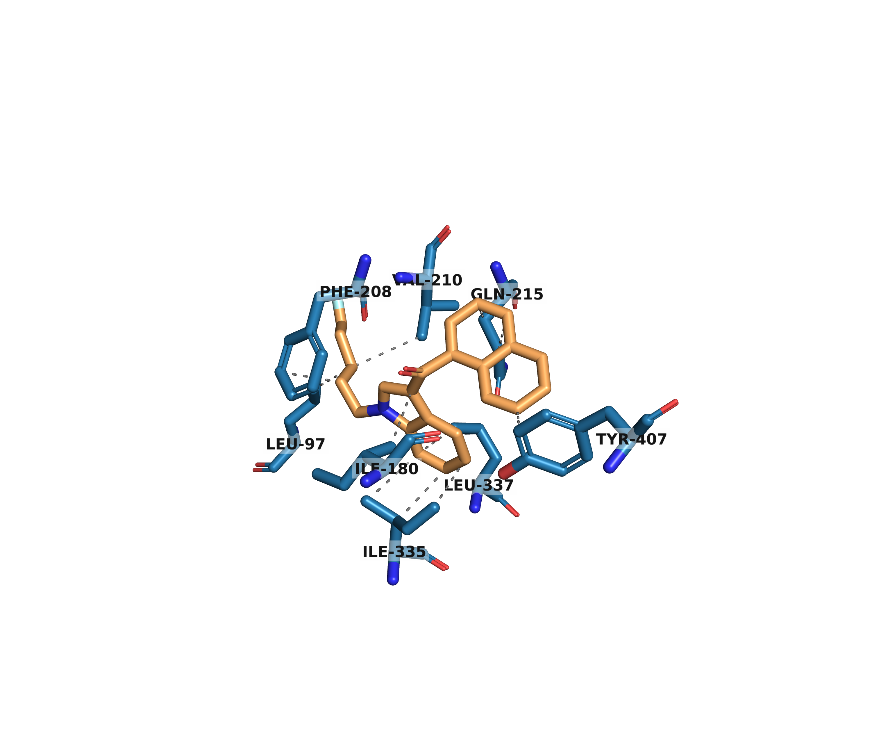

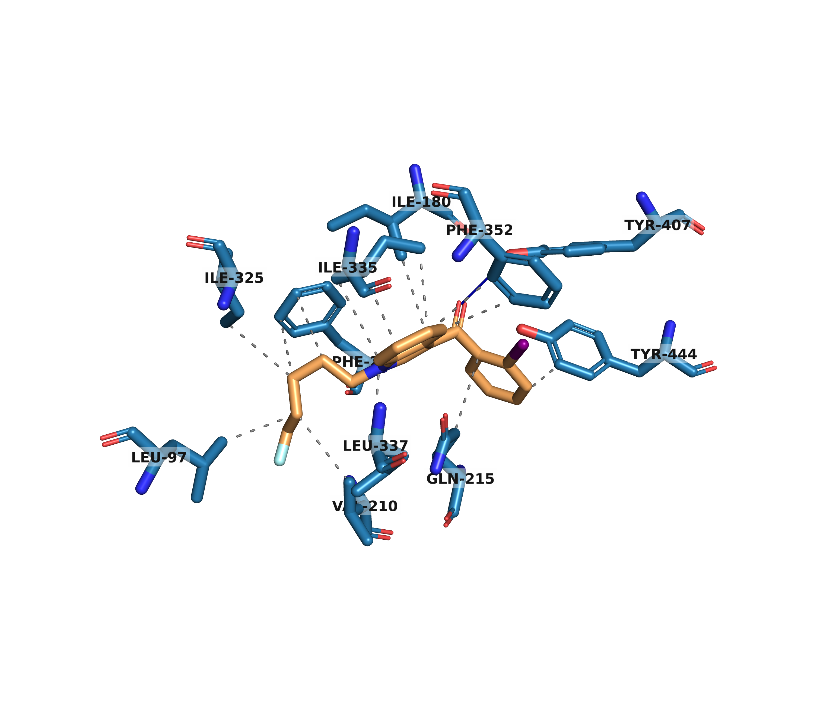


**AM-694 (5) in MAO-A**

**1-(5-fluoropentyl)-1*H*-Indole (6) in MAO-A**


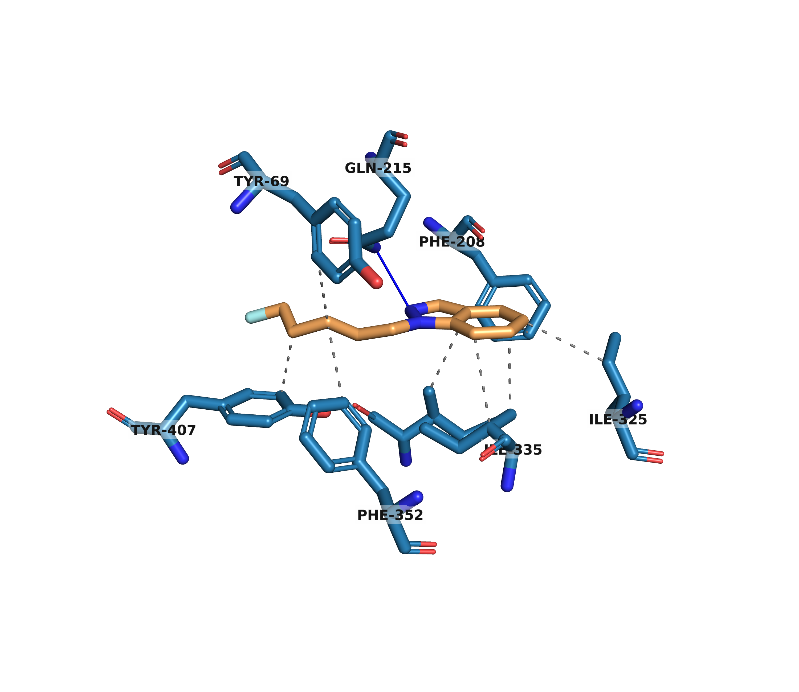


**1-(5-fluoropentyl)-1*H*-Indazole (7) in MAO-A**

**1-pentyl-1*H*-Indole (8) in MAO-A**


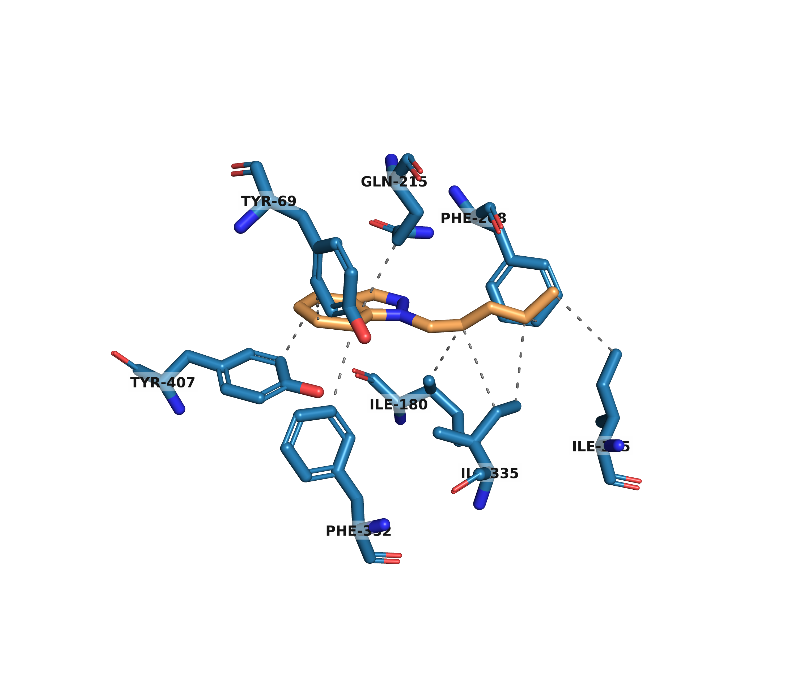


**1-pentyl-1*H*-Indazole (9) in MAO-A**


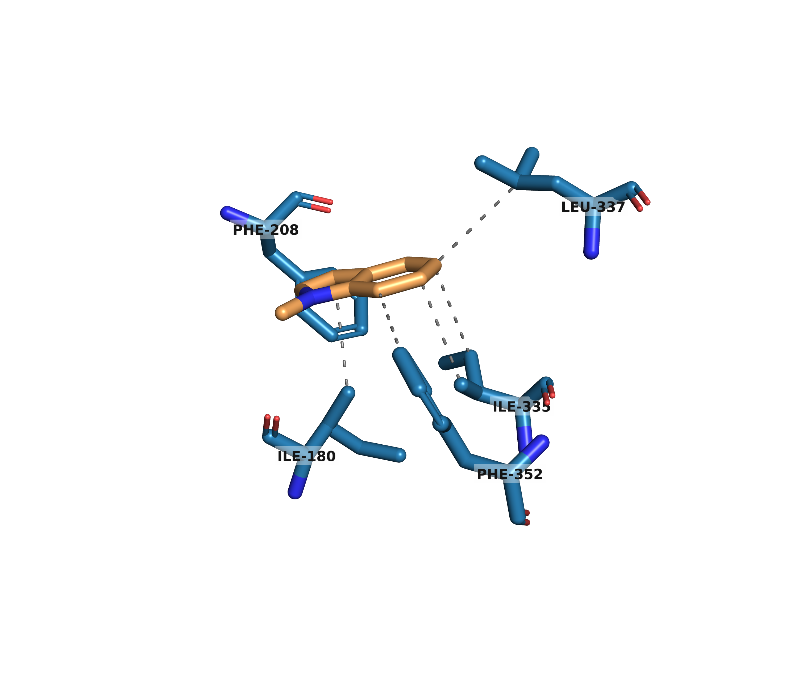


**1-methyl-1H-Indole (10) in MAO-A**


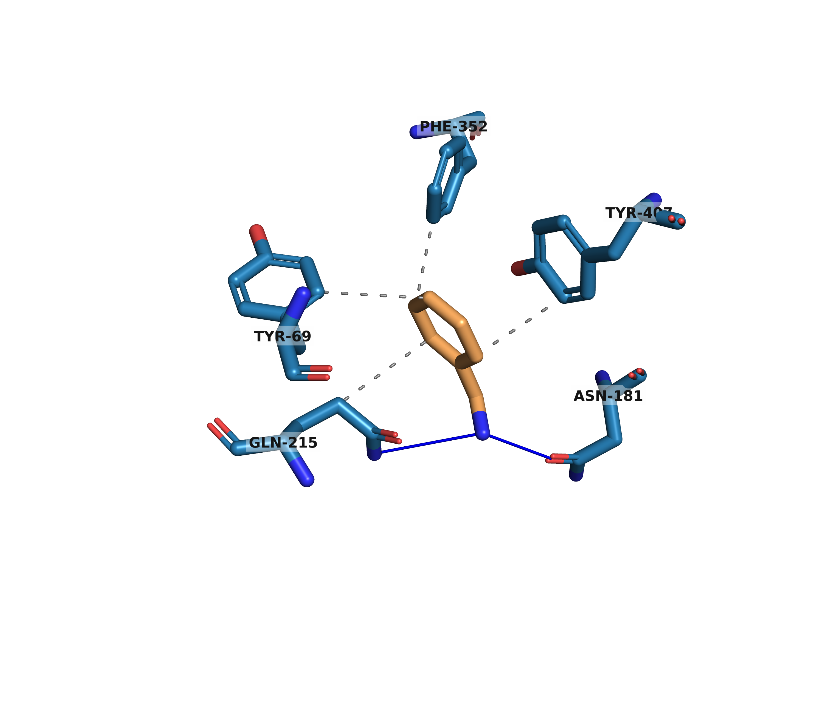


**Benzylamine (11) in MAO-A**


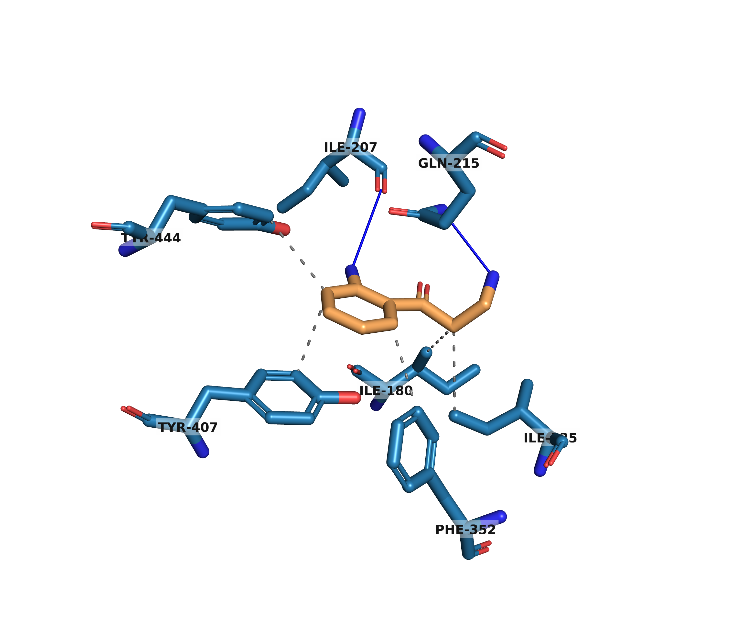


**Kynuramine (12) in MAO-A**


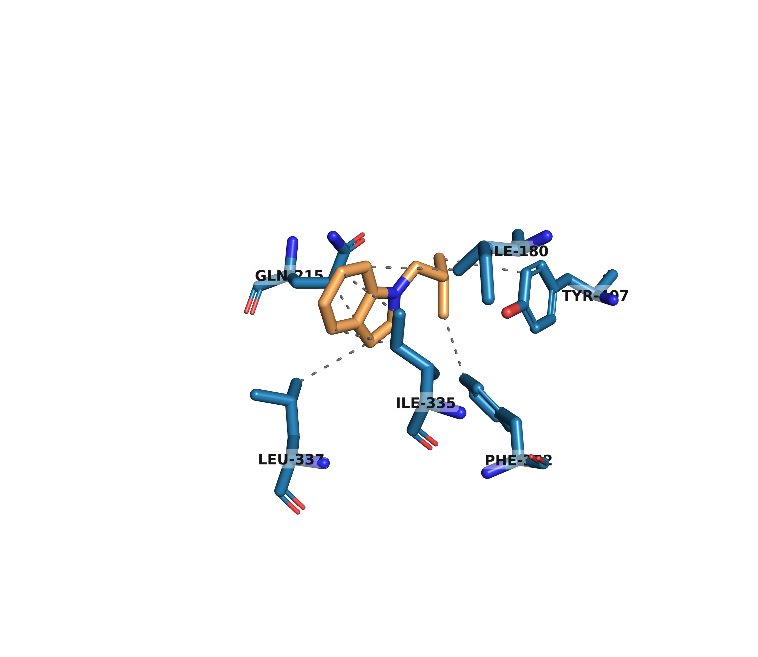

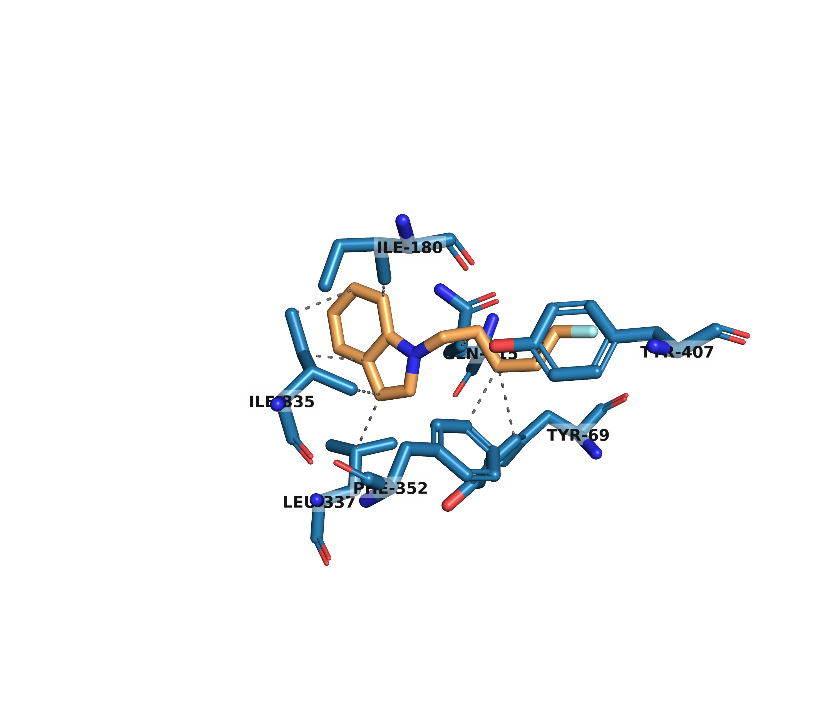


**Figure S2.** Lowest energy binding poses between ligands and residues in the active site of MAO-A. Structure figures were generated using PyMol (The PyMOL Molecular Graphics System, Version 2.4.1, Schrödinger, LLC).

**5F-ADB (1) in MAO-B**


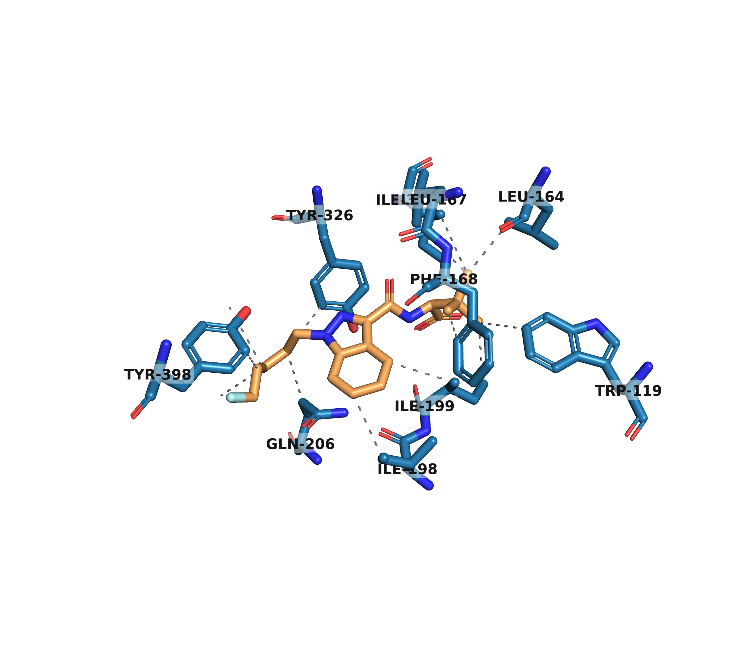


**5F-MDMB-PICA (2) in MAO-B**


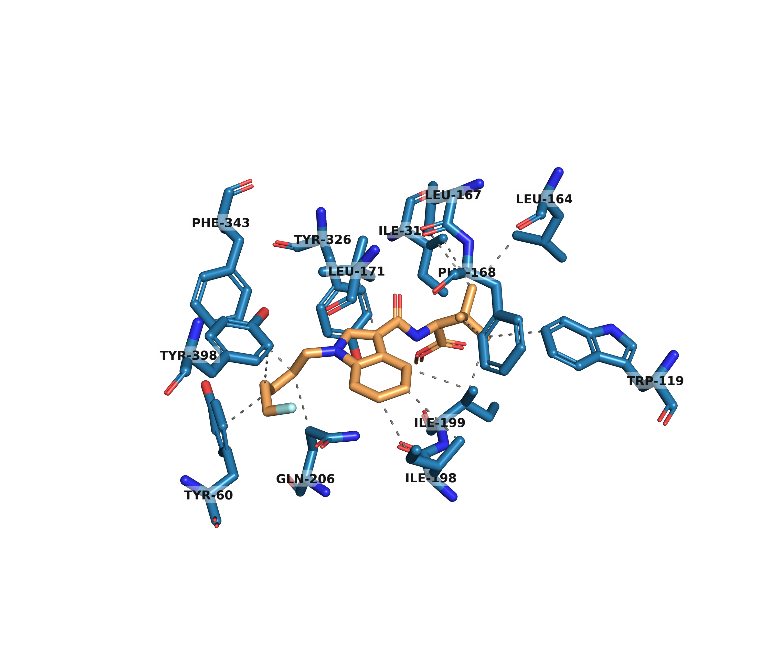


**AM-2201 (4) in MAO-B**


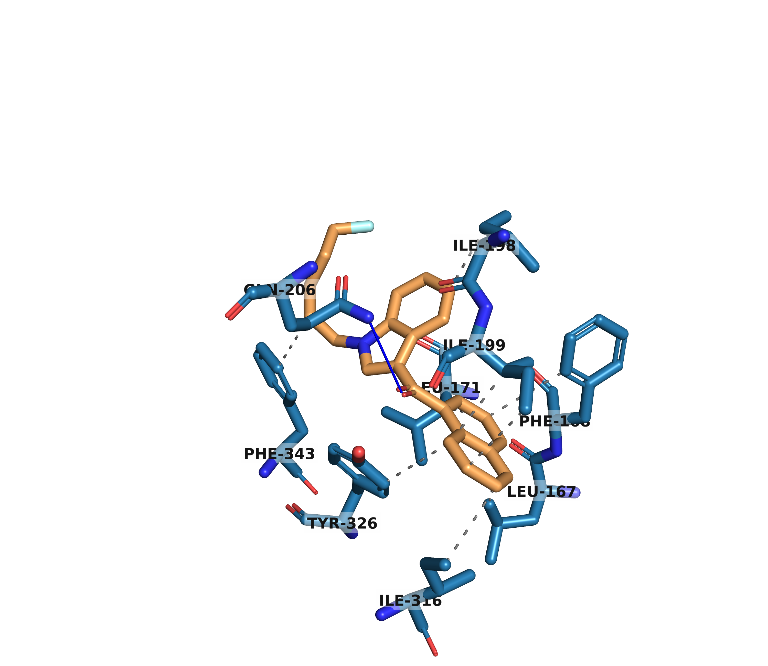


**AM-694 (5) in MAO-B**


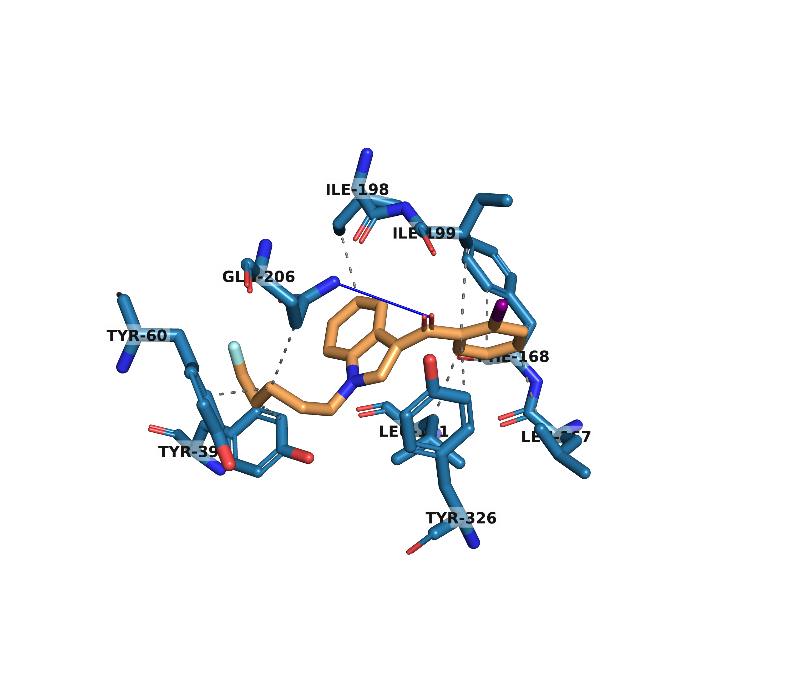


**1-(5-fluoropentyl)-1*H*-Indole (6) in MAO-B**


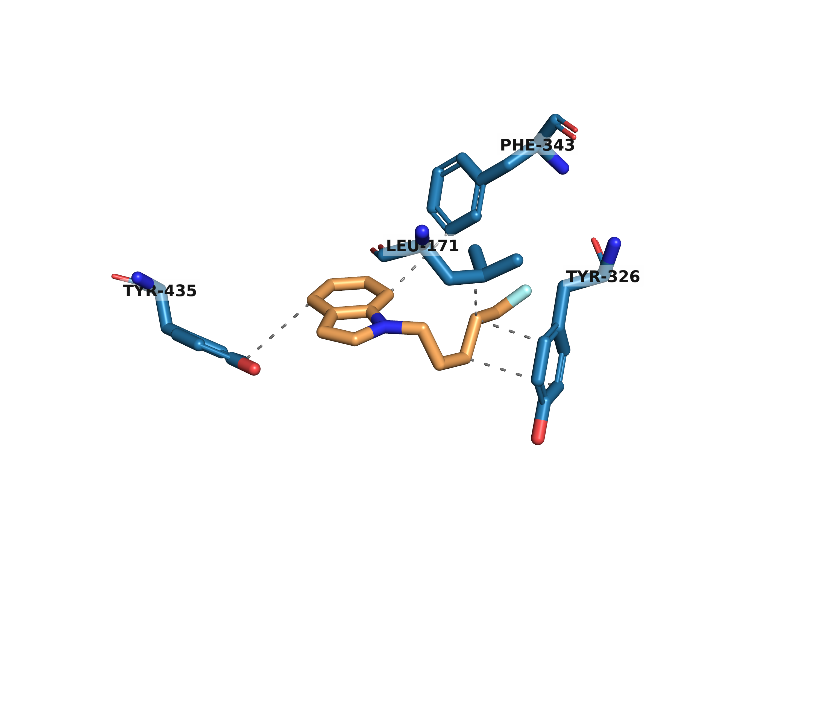


**1-(5-fluoropentyl)-1*H*-Indazole (7) in MAO-B**


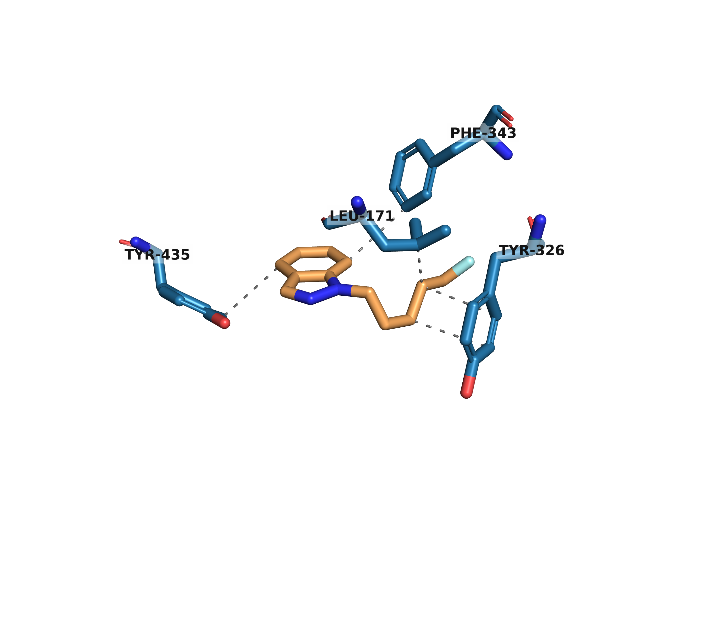


**1-pentyl-1H-Indole (8) in MAO-B**


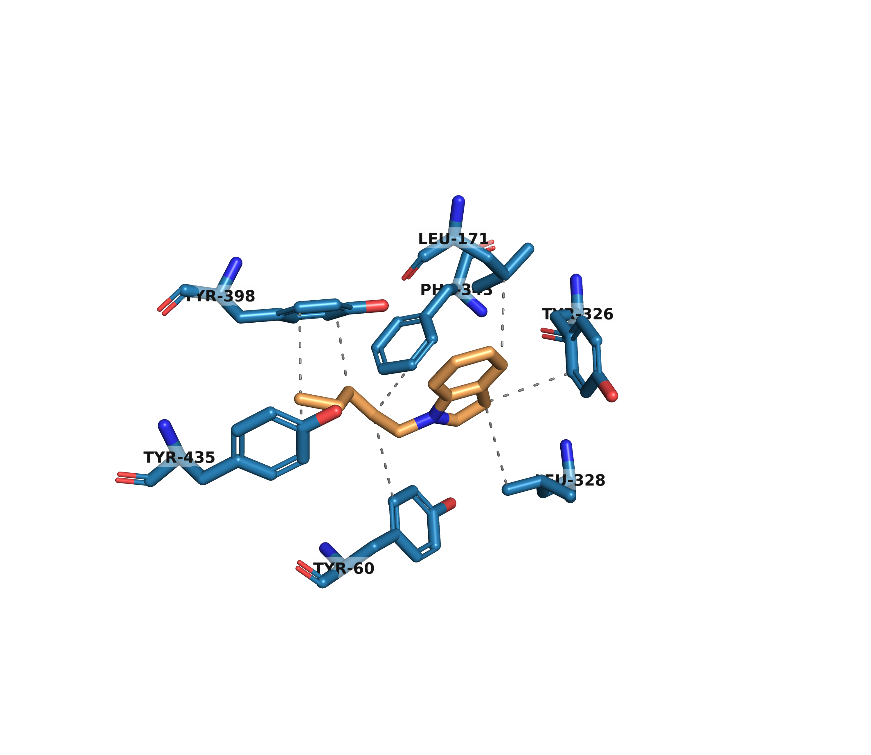


**1-pentyl-1*H*-Indazole (9) in MAO-B**


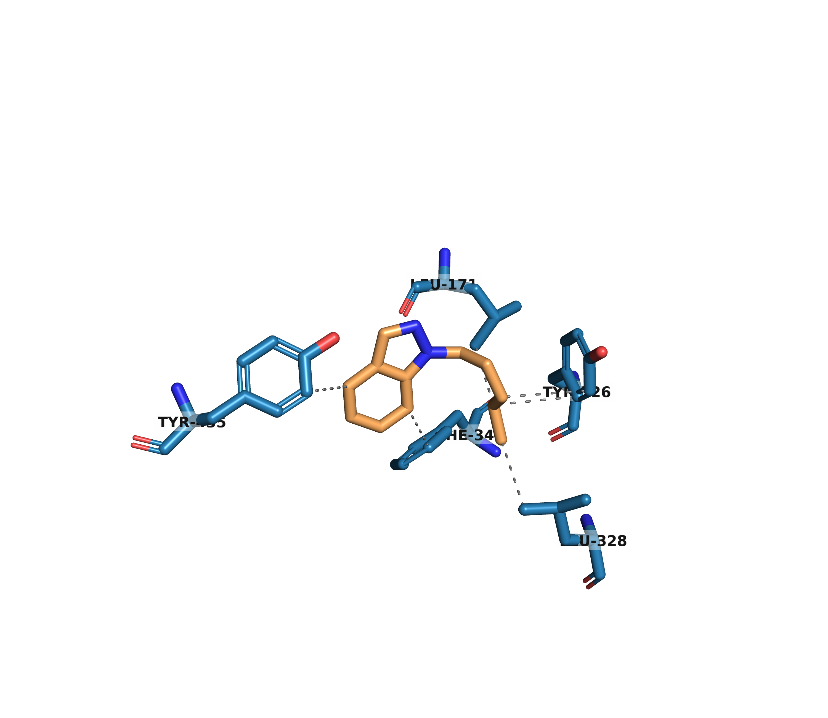


**1-methyl-1H-Indole (10) in MAO-B**


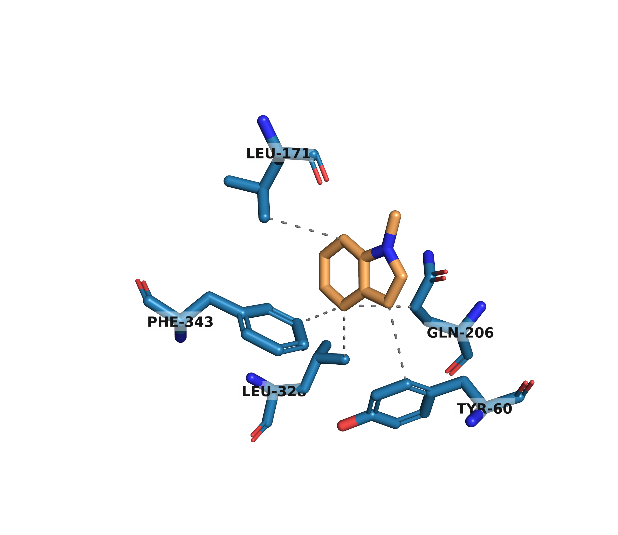


**Benzylamine (11) in MAO-B**


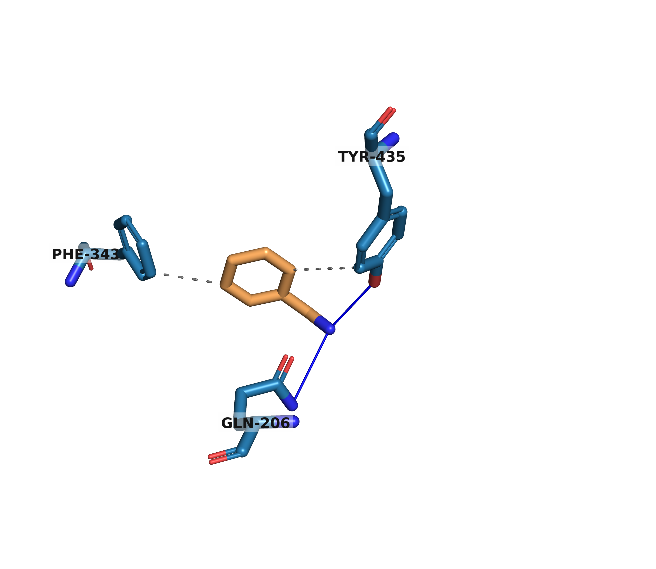


**Kynuramine (12) in MAO-B**


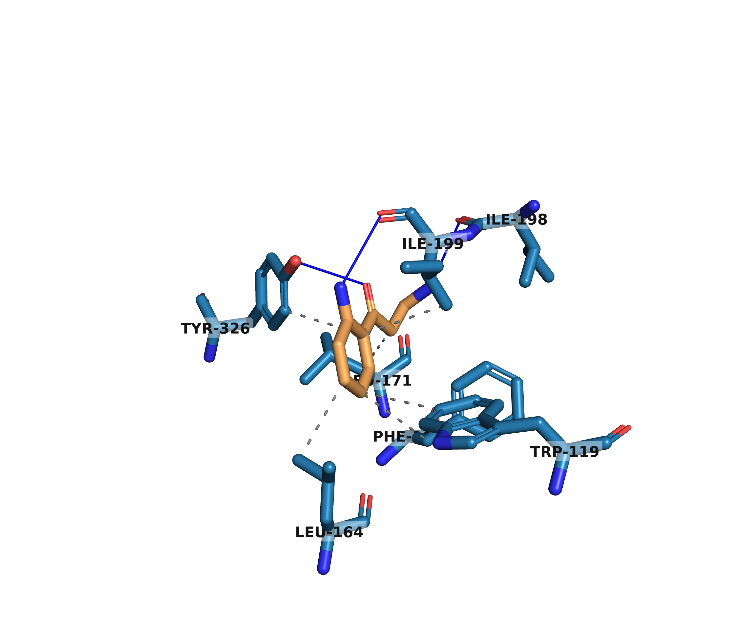


**Figure S3.** Lowest energy binding poses between ligands and residues in the active site of MAO-B. Structure figures were generated using PyMol (The PyMOL Molecular Graphics System, Version 2.4.1, Schrödinger, LLC).


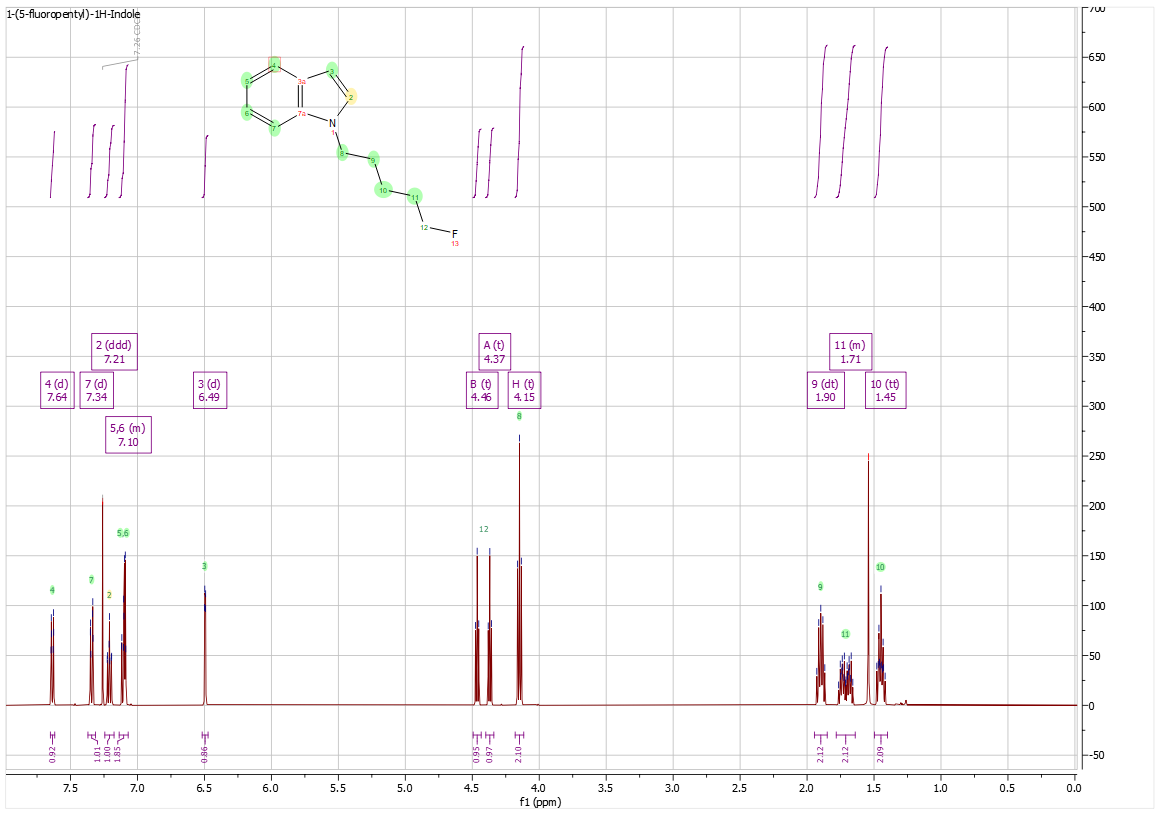


**Figure S4.** ^1^H NMR for N-5-fluoropentylindole, **6**.


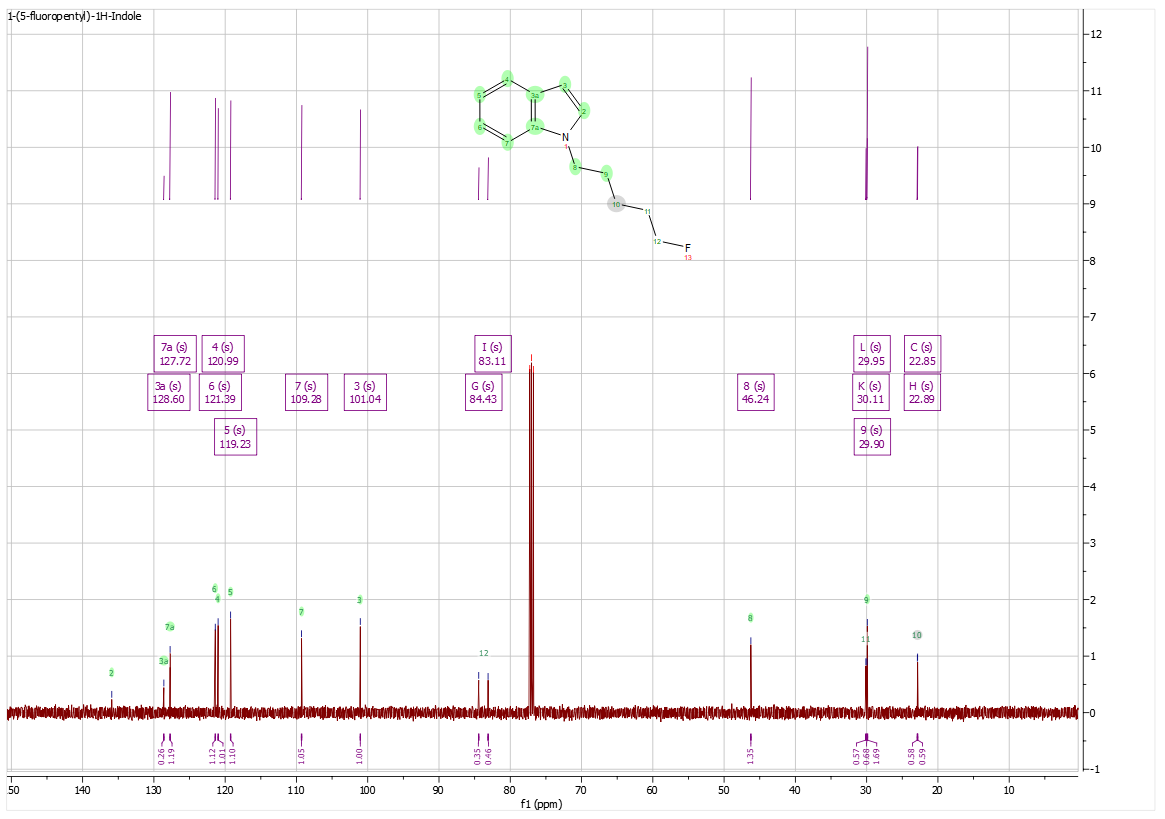


**Figure S5.** ^13^C NMR for N-5-fluoropentylindole, **6**.


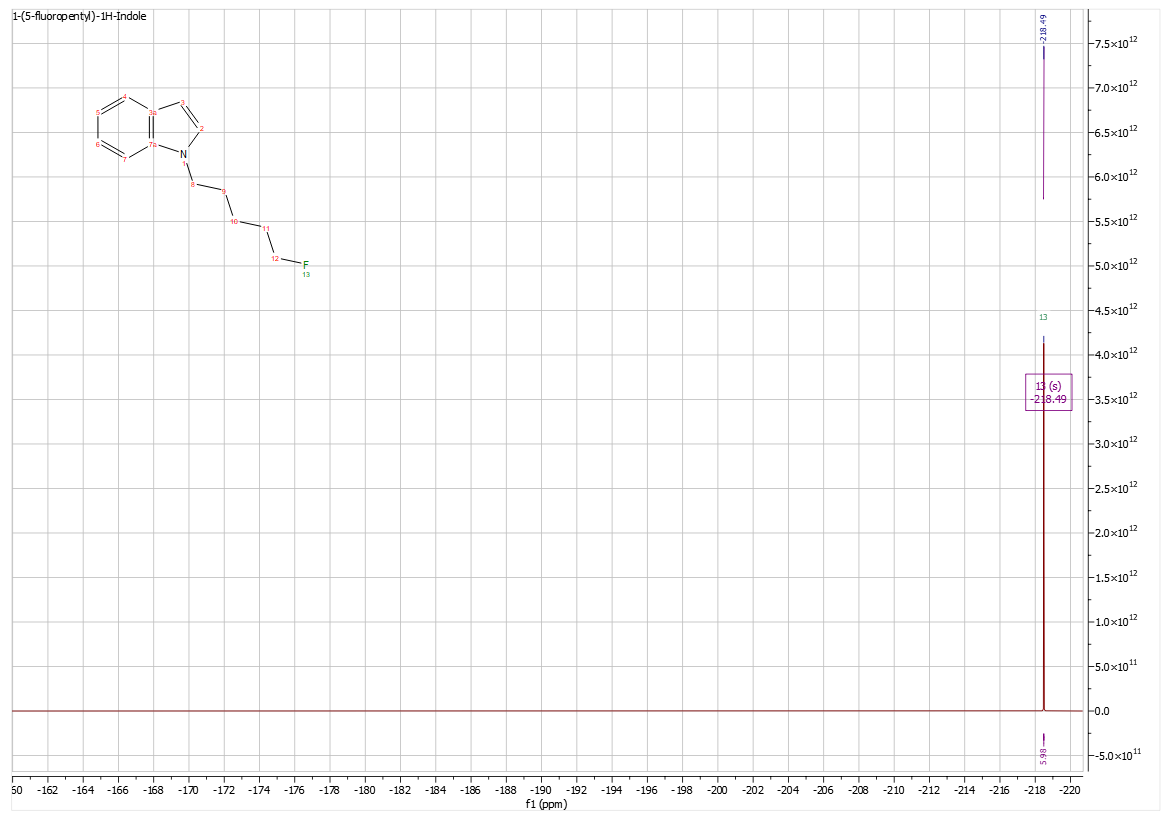


**Figure S6.** ^19^F NMR for N-5-fluoropentylindole, **6**.

**Figure S7.** IR spectrum for N-5-fluoropentylindole, **6**.


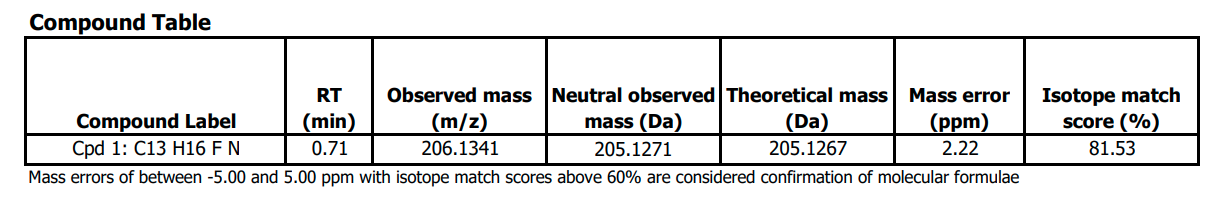


**Figure S8.** MS confirmation for N-5-fluoropentylindole, **6**.


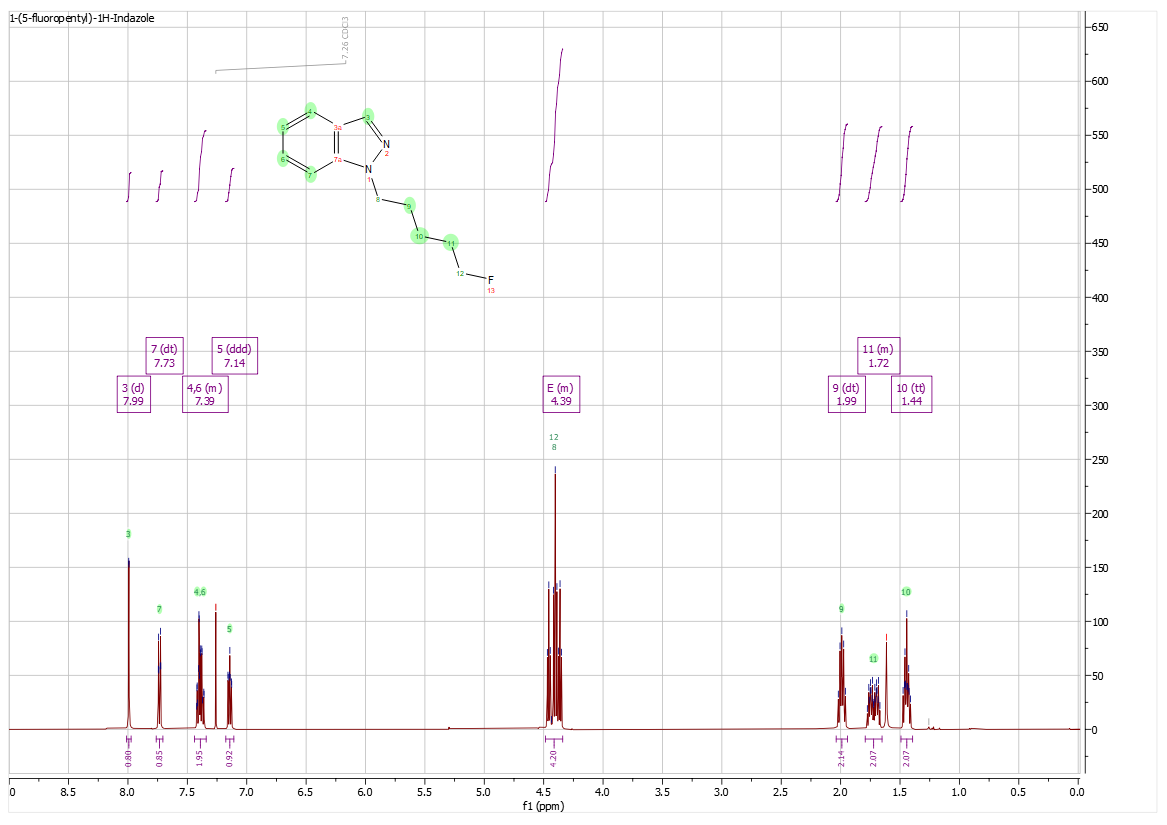


**Figure S9.** ^1^H NMR for N-5-fluoropentylindazole, **7.**


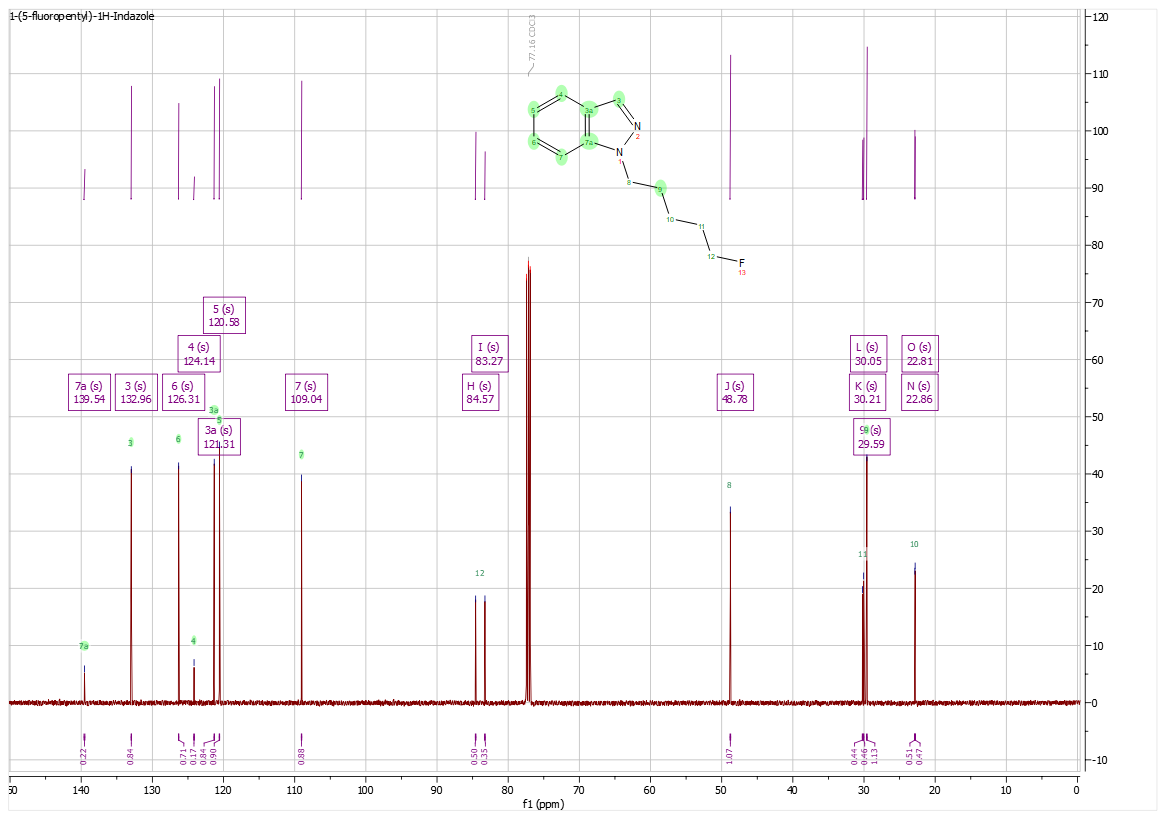


**Figure S10.** ^13^C NMR for N-5-fluoropentylindazole, **7**.


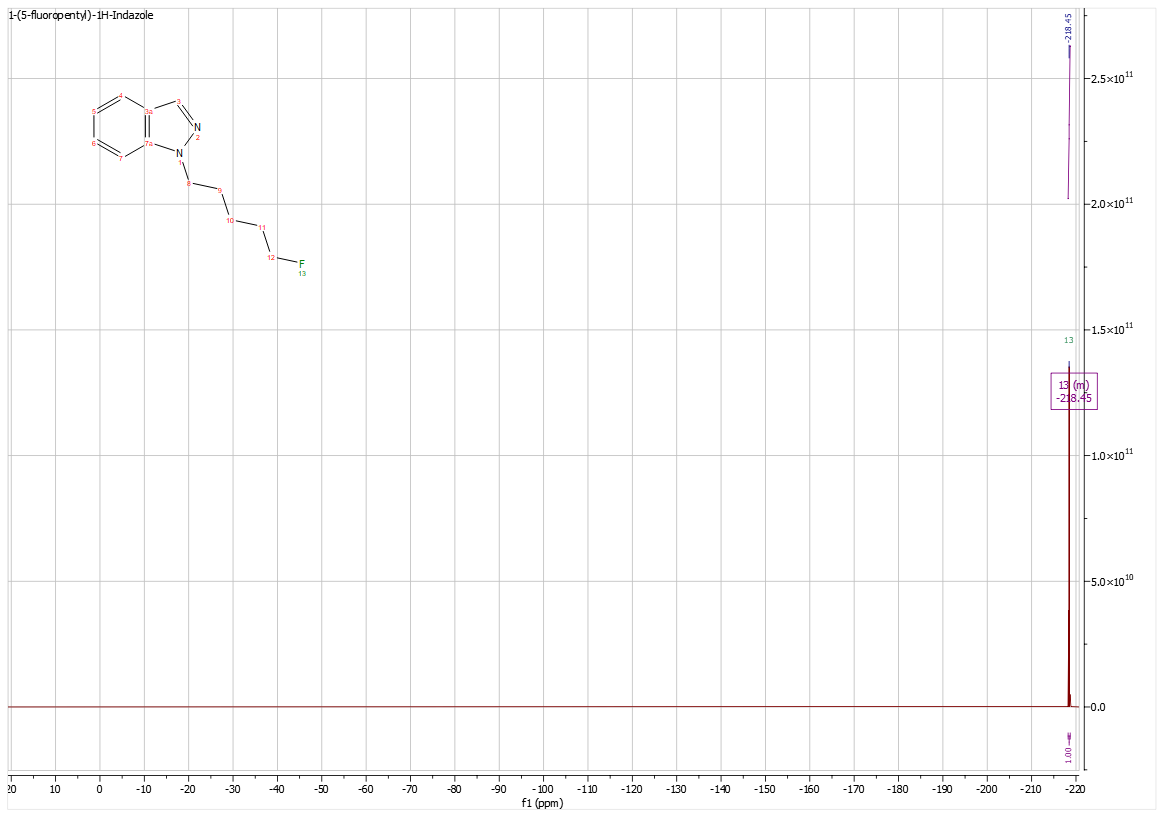


**Figure S11.** ^19^F NMR for N-5-fluoropentylindazole, **7**.

**Figure S12.** IR spectrum for N-5-fluoropentylindazole, **7**.


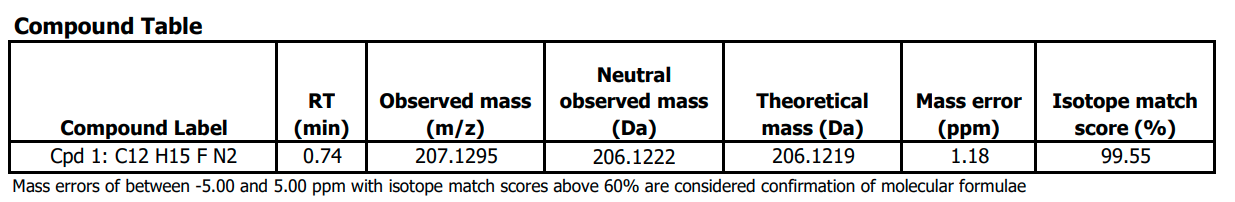


**Figure S13.** MS confirmation for N-5-fluoropentylindazole, **7**.


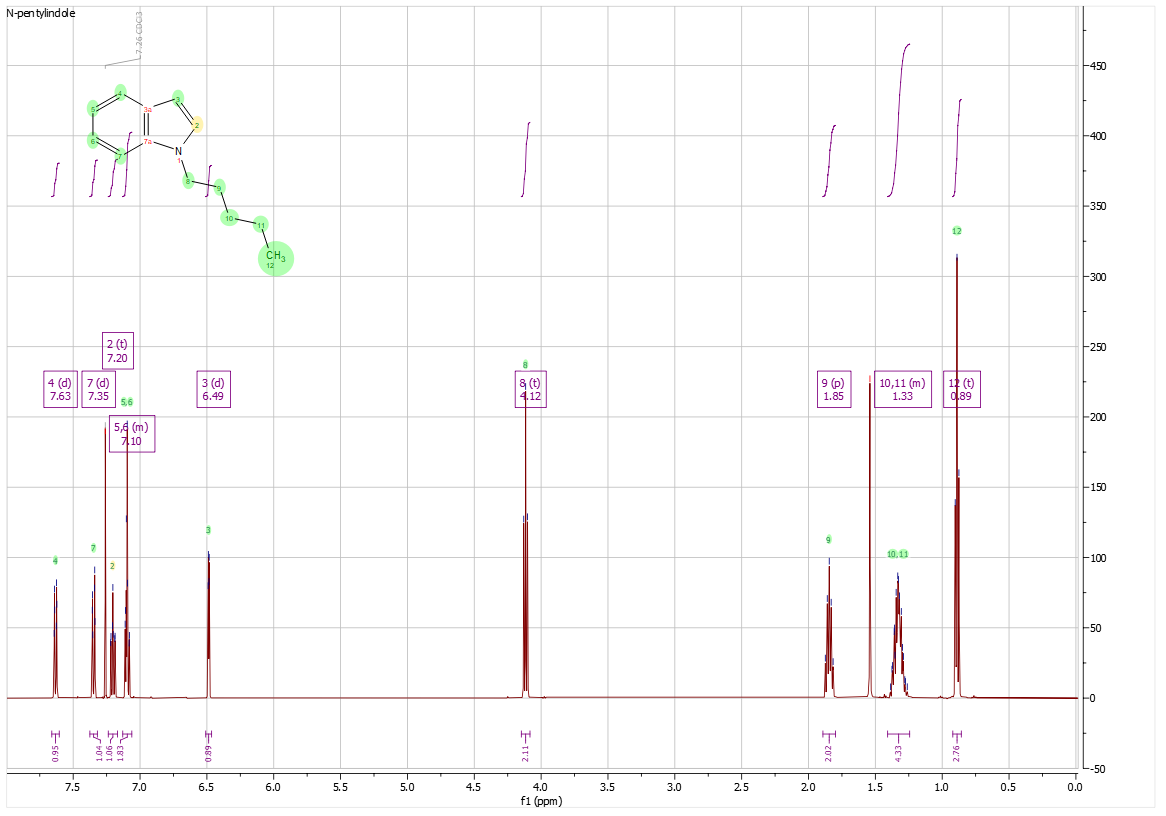


**Figure S14.** ^1^H NMR for N-pentylindole, **8.**


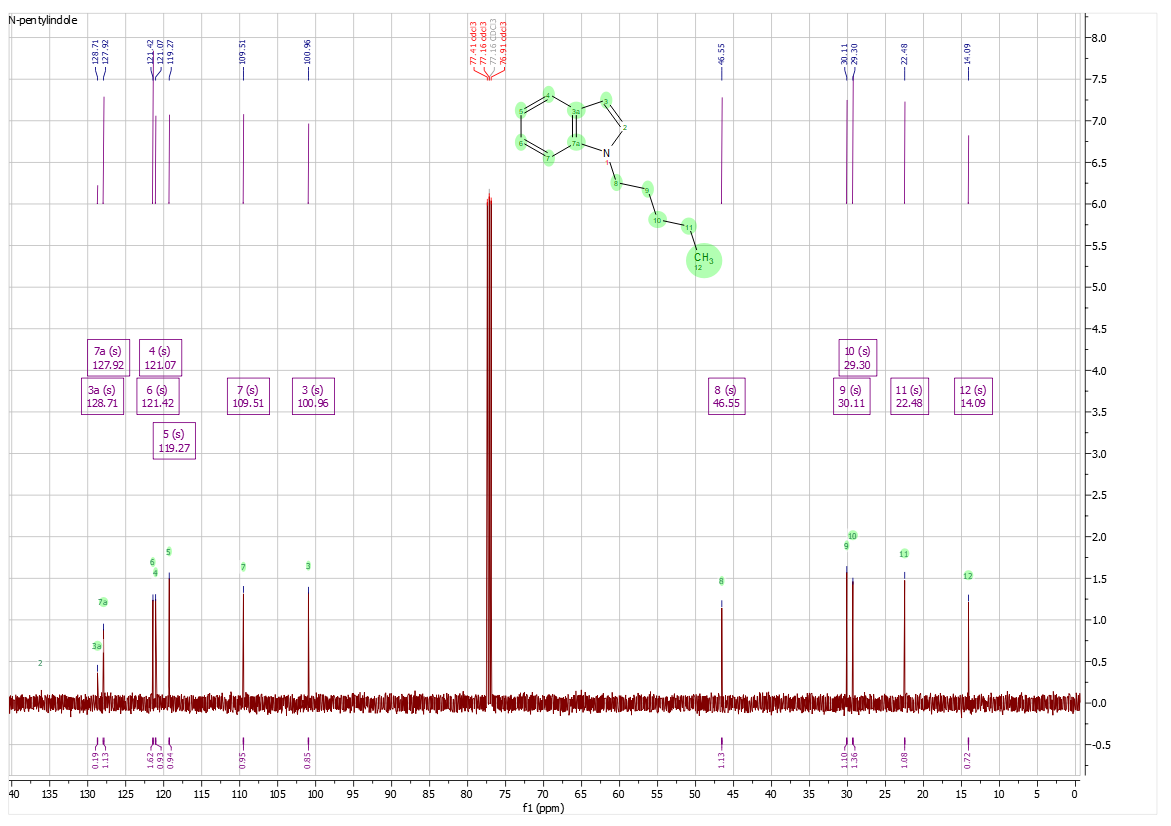


**Figure S15.** ^13^C NMR for N-pentylindole, **8.**

**Figure S16.** IR spectrum for N-pentylindole, **8.**


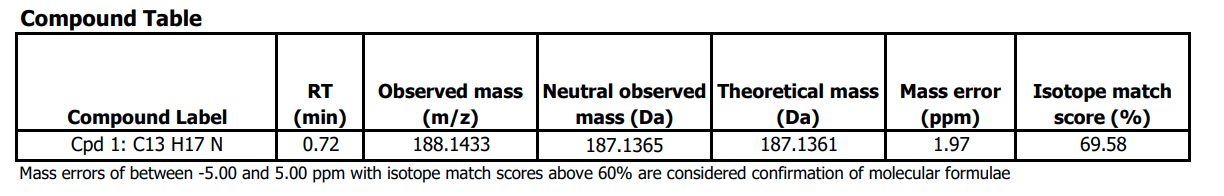


**Figure S17.** MS confirmation for N-pentylindole, **8.**


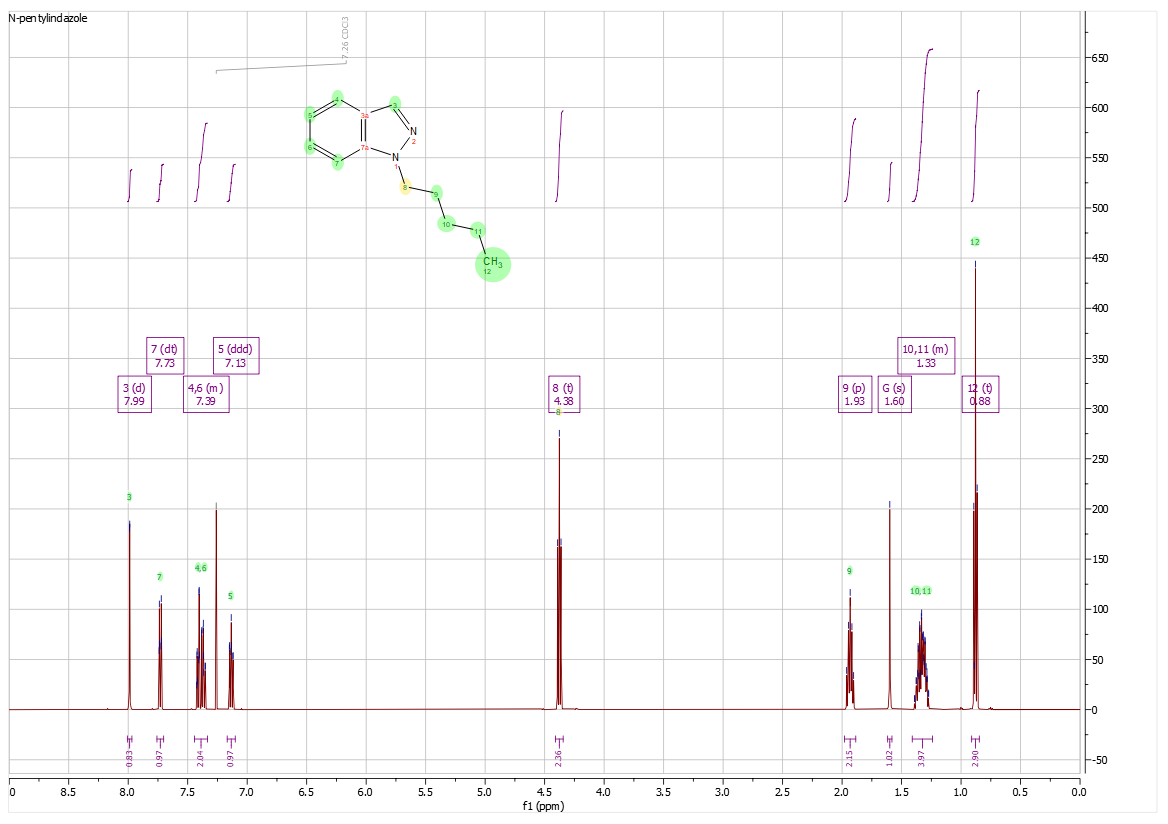


**Figure S18.** ^1^H NMR for N-pentylindazole, **9.**


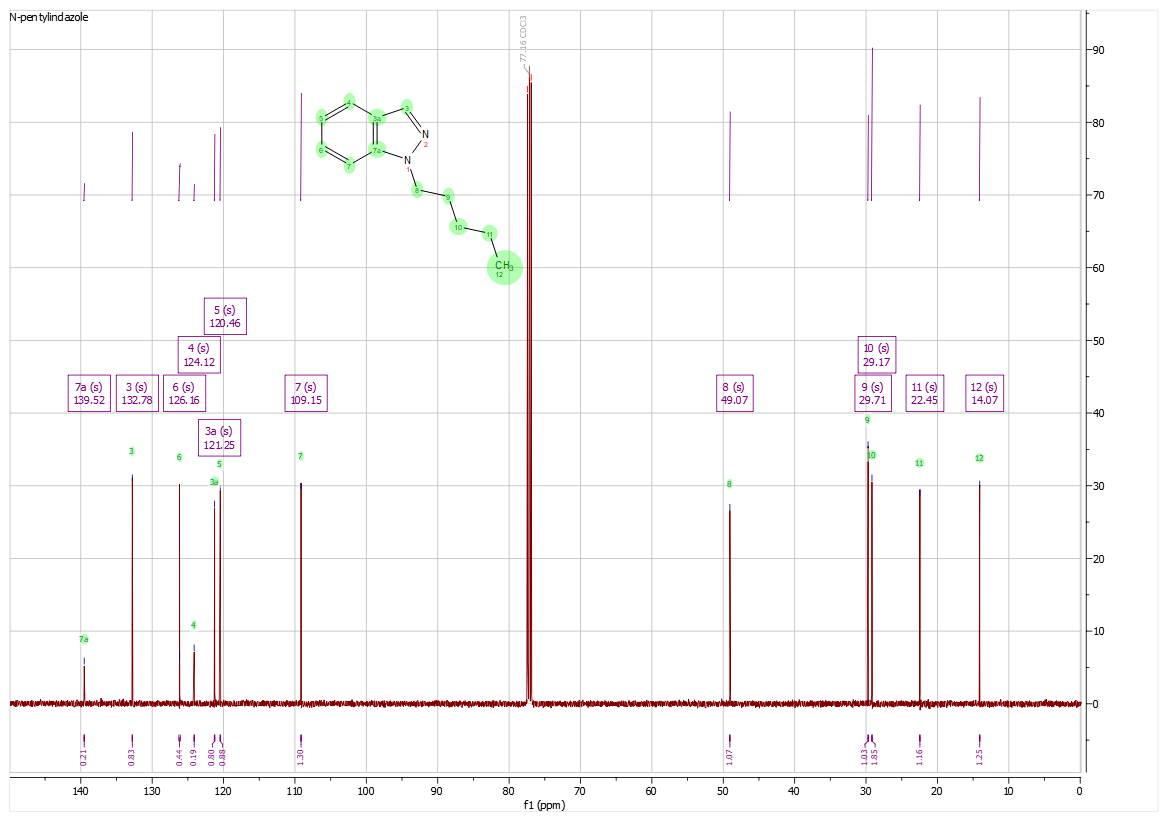


**Figure S19.** ^13^C NMR for N-pentylindazole, **9.**

**Figure S20.** IR spectrum for N-pentylindazole, **9.**


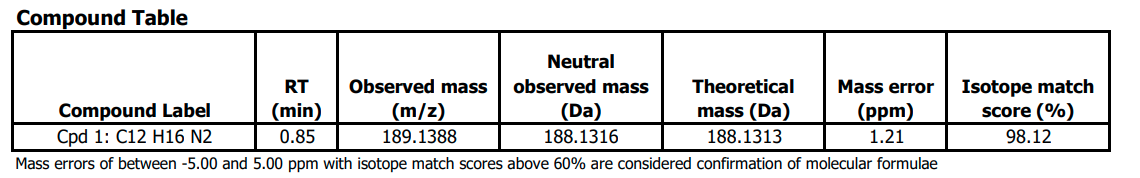


**Figure S21.** MS confirmation for N-pentylindazole, **9.**
